# Climatic, Socioecological and Environmental Determinants of *Aedes* spp. Dynamics at the Community Interface: A Systematic Review With Reflections From a One Health Perspective

**DOI:** 10.1111/tmi.70131

**Published:** 2026-03-24

**Authors:** Ana Izabel Passarella Teixeira, Giovanna Rotondo de Araújo, Diogo Tavares Cardoso, David Soeiro Barbosa, Christina Pettan‐Brewer, Joaquim Pinto Nunes Neto, Ruana Renosto Delai, Cláudia Turra Pimpão, Lívia Medeiros Neves Casseb

**Affiliations:** ^1^ Universidade Federal do Mato Grosso do Sul Paranaíba Mato Grosso do Sul Brazil; ^2^ Instituto Evandro Chagas Ananindeua Pará Brazil; ^3^ Universidade Federal de Minas Gerais Belo Horizonte Minas Gerais Brazil; ^4^ School of Medicine University of Washington Seattle Washington USA; ^5^ Pontifícia Universidade Católica do Paraná Curitiba Paraná Brazil

**Keywords:** *Aedes*, climate change, one health, social determinants, vector ecology

## Abstract

**Background and Objectives:**

Mosquitoes of the genus *Aedes,* including but not limited to *Aedes aegypti* , are major vectors of arboviruses such as dengue, Zika and other related diseases. Their global expansion is driven by climate change, globalisation, urbanisation and human mobility. Understanding how these factors shape their reproduction, survival and persistence is crucial for strengthening integrated vector control within a One Health framework. This review synthesises current evidence on these determinants to support more sustainable control strategies.

**Methods:**

A systematic review was conducted following PRISMA guidelines across PubMed/MEDLINE and SciELO through June 2025. Search strategies combined terms related to *Aedes* spp., environmental, climatic, socio‐ecological determinants and One Health. Eligible studies examined factors influencing reproduction, survival or persistence of any Aedes species under natural or semi‐natural conditions. Case reports, clinically focused studies and laboratory experiments lacking ecological realism were excluded. Two reviewers independently screened records and extracted data. Findings were synthesised descriptively.

**Results:**

From 1007 records screened, 98 were included. Most were conducted in Brazil (18.4%), China (12.2%) and Argentina (11.2%). Ecological–epidemiological studies (30.6%) and environmentally oriented laboratory simulations (24.5%) were predominant. The evidence highlighted: (i) microenvironmental factors—such as water quality, nutrient availability, pollutants, and artificial light—affecting larval development and vector competence; (ii) climatic variables, especially temperature, rainfall, and humidity, as major drivers of mosquito abundance and transmission potential; (iii) urbanisation, inadequate housing, socioeconomic vulnerability, and irregular water supply as key amplifiers of infestation; and (iv) widespread insecticide resistance and the limited impact of conventional larvicides as major barriers to control.

**Conclusions:**

Environmental, climatic and socio‐ecological determinants critically shape *Aedes* persistence and arbovirus transmission. Integrating these determinants into surveillance and vector control programs through a One Health approach enables earlier detection, more targeted interventions and the development of sustainable strategies to reduce the global burden of arboviral diseases.

## 1 Introduction

Aedes mosquitoes—especially *Aedes aegypti* and *Aedes albopictus* —are the main vectors of dengue and other arboviruses. Their global expansion is fuelled by climate change, globalisation, rapid urbanisation, and increased human mobility, driving unprecedented arboviral transmission, including the record dengue incidence reported in 2024 [1, 2]. Key eco‐behavioural traits—especially the strong anthropophilic nature of *Ae. aegypti* and the shared exploitation of artificial containers in dense urban areas—significantly boost Aedes vectorial capacity. Although climate changes in their current and future ranges, human connectivity (migration, trade, transportation) and urbanisation are the primary drivers of their expansion. Without strengthened entomological surveillance and targeted vector‐control strategies, reducing the growing global burden of Aedes‐borne arboviruses will remain unattainable [1, 3].

Effective mosquito control is challenging due to *Aedes'* adaptive life cycle and sensitivity to human‐driven environmental and climatic conditions. A One Health approach strengthens interventions by integrating ecological, animal and human factors with public health surveillance, enabling coordinated monitoring and earlier outbreak detection [3, 4]. To address this gap, we conducted a PRISMA‐guided systematic review synthesising evidence on determinants of Aedes mosquito reproduction and persistence. The PI/ECO‐based research question was: Which environmental, climatic and socio‐ecological factors influence Aedes reproduction, breeding‐site productivity and environmental persistence across diverse ecological contexts within an integrated One Health framework?

## 2 Methods

### 2.1 Type of Study and Reporting Guidelines

A systematic literature review was conducted with a qualitative, exploratory approach following the PRISMA 2020 statement [5]. The review aimed to synthesise evidence on climatic, environmental and socio‐ecological determinants associated with the persistence, survival and population dynamics of *Aedes* spp., incorporating reflections under a One Health perspective.

### 2.2 Information Sources and Search Strategy

Searches were performed in Medline via PubMed and SciELO. The time frame included publications from January 2000 to June 2025. No language restrictions were applied. The final search strategies were adapted to the syntax of each database and conducted on the following dates: PubMed (June 3, 2025) and SciELO (June 9, 2025).

The complete search strategies, including full Boolean strings, database‐specific fields and number of records retrieved per query, are provided in Supporting Information (Table S1) to ensure transparency and reproducibility.

### 2.3 Study Selection Process

**FIGURE 1:**
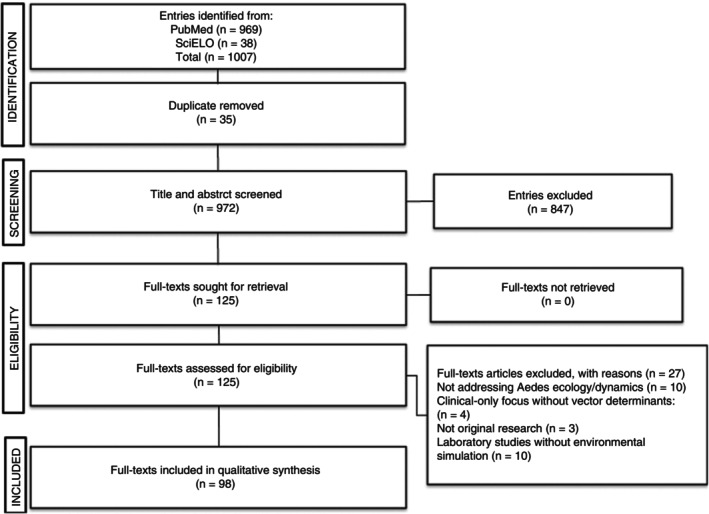
PRISMA 2020 flow diagram of the study selection process.

Articles classified as “included” or “unclear” after the first full‐text assessment underwent a second round of full‐text review to confirm eligibility. The selection process and the number of records at each stage are presented in the PRISMA flow diagram (Figure 1), including reasons for exclusion in the full‐text stage.

### 2.4 Eligibility Criteria

*Inclusion criteria*: Original research articles addressing climatic, environmental, and/or socio‐ecological determinants related to the reproduction, survival, persistence, abundance, or population dynamics of *Aedes* spp.; studies conducted in tropical and subtropical regions; laboratory studies simulating natural conditions to evaluate climatic variables; and studies adopting either an explicit One Health approach or an interdisciplinary perspective (integration of environmental and human health components, and where applicable, animal‐related aspects such as host presence, wildlife interfaces, or zoonotic surveillance contexts).

*Exclusion criteria*: Case reports, letters to the editor, conference abstracts, and purely clinical studies of arboviruses without linkage to *Aedes* mosquitoes' genus ecology or environmental determinants; laboratory studies limited to sterilisation or infection control procedures without environmental simulation; and duplicate records.

*Full‐text retrieval*: Articles for which the full text could not be retrieved after reasonable efforts (including institutional access, online databases and author‐contact attempts when applicable) were documented as “not retrieved” and excluded due to inability to extract essential methodological and outcome data.

### 2.5 Data Extraction and One Health Operationalisation

A standardised extraction form was used to collect the following information: journal name; authors; publication year; study design; epidemiological classification (when applicable); study setting and country/region; objectives; climatic determinants; environmental determinants; socio‐ecological/urban determinants; analytical methods; main outcomes; key findings; study limitations; and relevant comments.

To strengthen the One Health perspective, each study was additionally coded according to the presence of environmental components (e.g., climate, land use, breeding sites, sanitation) or human‐related components (e.g., community behaviours, housing, infrastructure, surveillance systems) and animal‐related or ecological interface components when present (e.g., domestic animals, wildlife interface, host availability, ecosystem interactions).

The level of integration across these domains was categorised as descriptive, analytical or intersectoral (i.e., evidence explicitly supporting integrated surveillance, multisectoral interventions or policy‐level recommendations).

Extraction was performed independently and by two reviewers. Duplicate articles were identified and excluded using Zotero software [6]. Disagreements were resolved by consensus or third reviewer adjudication.

### 2.6 Data Synthesis and Thematic Organisation

Findings were synthesised narratively and organised into three thematic axes derived from determinants reported across studies: (i) microenvironmental and chemical shifts in *Aede*s breeding sites; (ii) climatic variables as drivers of *Aedes* ecology and population dynamics (including ambient temperature); and (iii) urbanisation and human practices modulating *Aedes* populations. To enhance interpretability and minimise overlap across findings, determinants were further consolidated into climatic, environmental and socio‐ecological domains, with emphasis on the most recurrent and policy‐relevant factors and representative examples from included studies.

### 2.7 Geographical Analysis

The geographical distribution of the included studies was assessed at the country level based on the study setting reported by the authors. Each study was assigned to one country whenever a single‐country setting was reported. Studies conducted in more than one country were classified as multicountry and were excluded from the choropleth map to prevent double counting and to avoid misinterpretation of national totals. Spatial visualisation was generated using the Microsoft Excel map chart tool (Bing Maps cartographic platform) [7].

### 2.8 Protocol Registration

The review protocol was registered at the Open Science Framework (OSF) under code “5mfv6”.

## 3 Results

A total of 1007 records were identified across the initial and additional searches. After removal of 35 duplicates, 972 records underwent title and abstract screening, and 125 full‐text articles were assessed for eligibility. Among these, 27 studies (21.6%) were excluded at the full‐text stage for not meeting the inclusion criteria. Ultimately, 98 studies (78.4%) were included in the qualitative synthesis. The complete selection process is presented in Figure 1 (PRISMA flow diagram).

**FIGURE 2:**
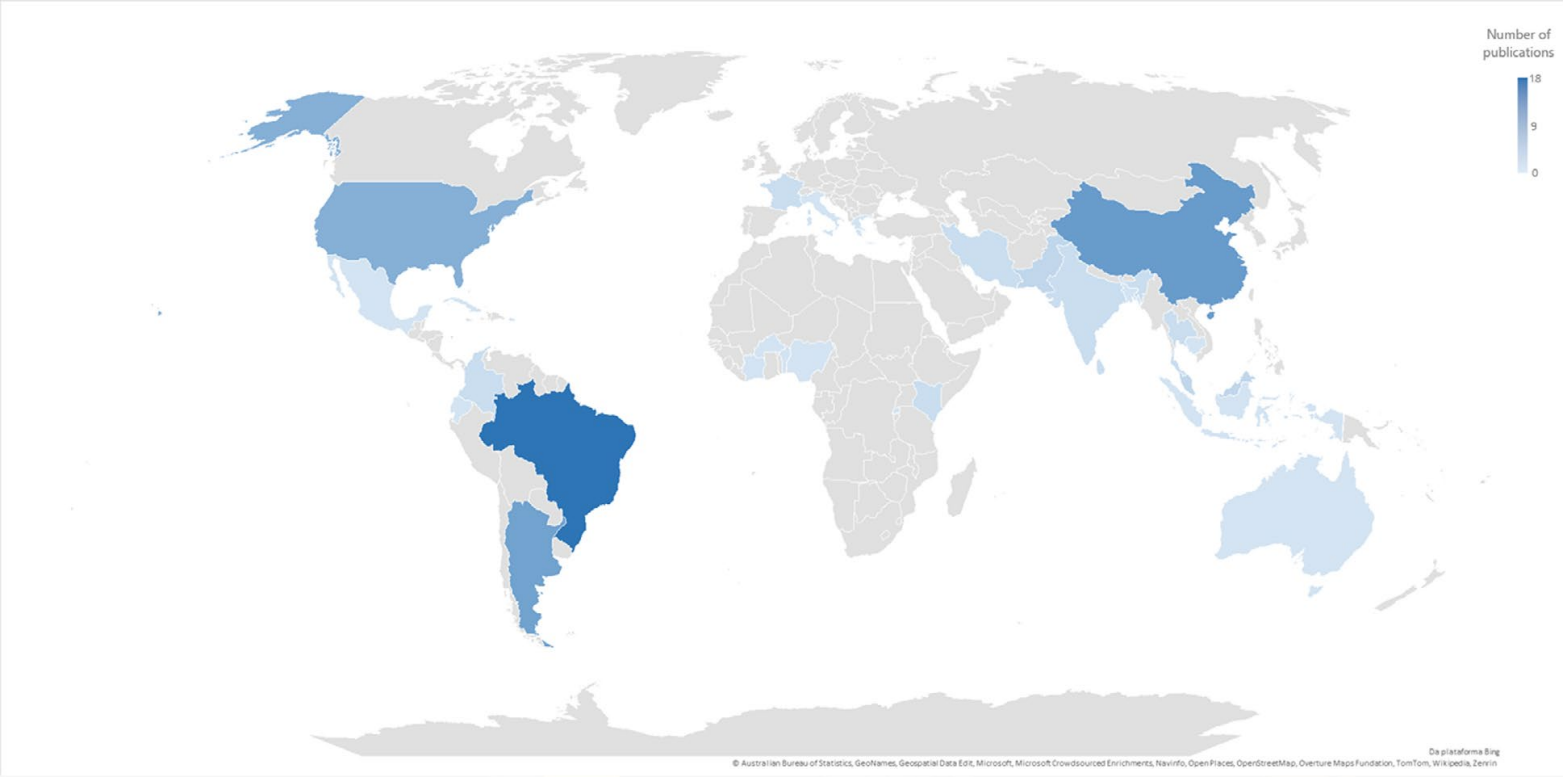
Global distribution of the research included in this review (single‐country studies only *n* = 89).

The geographic analysis of the 98 included studies showed a heterogeneous global distribution. Most studies were conducted in Brazil (18; 18.4%), followed by China (12; 12.2%) and Argentina (11; 11.2%). The United States accounted for 9 studies (9.2%), and Puerto Rico contributed 2 additional studies (2.0%). Other countries with multiple studies included Malaysia and Pakistan (3 studies each), and Colombia, France, India, Iran, Kenya, Singapore, Sri Lanka, and Thailand (2 studies each). Countries represented by a single study included Australia, Bangladesh, Benin, Burkina Faso, Cambodia, Côte d'Ivoire, Cuba, Ecuador, Greece, Indonesia, Italy, Mexico, Nigeria, Rwanda, and São Tomé and Príncipe. Multi‐country studies comprised 9 studies (9.2%) and were analysed separately; therefore, they were not attributed to individual countries in the country‐level map. Overall, the distribution indicates a concentration of research in Latin America and Asia, with additional contributions from Africa, Europe and Oceania, reflecting the broad endemic context of Aedes‐borne diseases. The country‐level spatial distribution of single‐country studies is presented in Figure 2.

Among the 98 studies, ecological–epidemiological investigations were the most frequent (30; 30.6%), relying on secondary data for predictive modelling and time‐series analyses to characterise arboviral patterns or identify areas suitable for increased *Aedes* abundance, with some validating predictions using trap‐derived entomological data. Laboratory‐based simulations constituted the second‐largest category (24; 24.5%), reproducing real‐world conditions under controlled settings, for example, assessing how microplastics influence mosquito reproduction and vector competence. Entomological field studies (17; 17.3%) involved the identification of eggs, larvae, pupae, and adults, occasionally integrating ecological or genetic analyses at local scales. Retrospective longitudinal studies (17; 17.3%) also used secondary datasets to model temporal relationships between environmental or epidemiological variables and arbovirus incidence or Aedes surveillance indicators.

Among the 98 studies analysed, ecological‐epidemiological investigations represented the largest proportion (30, 30.6%). These studies used secondary data for predictive modelling and time‐series analysis. They examined arbovirus case patterns or identified areas favourable for increased populations of mosquitos of the genus *Aede*s. Some studies also validated models using mosquito trap data. Laboratory‐based simulations were the next‐largest category (24, 24.5%). These examined real‐world scenarios under controlled conditions. For example, some studied how microplastics affect these arthropods' reproduction and viral transmission capacity [8]. Entomological studies (17, 17.3%) involved field identification of eggs, larvae, pupae and adults. Some combined this with ecological or genetic analyses at district or municipal levels. Retrospective longitudinal studies (17, 17.3%) used secondary data, modelled over time using time‐series or mathematical approaches. They assessed how certain variables influenced arbovirus cases or *Aedes* mosquitoes' surveillance indices.

Genetic studies represented 4.1% (4) and focused on mosquito or viral genetics. Cross‐sectional investigations accounted for 3.1% (3), assessing arbovirus seroprevalence and associated risk factors, including exposure to *Aedes* mosquitoes. Intervention studies (2; 2.0%) evaluated changes in mosquito populations before and after control measures, while one study (1.0%) provided a detailed description of an entomological surveillance system. Notably, 26.5% (26) of the studies included contributed to more than one thematic axis defined in the methodology.

### 3.1 Microenvironmental and Chemical Shifts in Mosquitoes of the Genus *Aedes* Breeding Locations

Addressing characteristics of the mosquito breeding microenvironment, 14 studies examined factors such as water quality and pollution, physicochemical parameters, solar incidence, and artificial light at breeding sites. All relevant studies are summarised in Table S2.

Water quality is a key determinant. Although *Ae. aegypti* typically prefers clean water [9], laboratory studies demonstrate substantial plasticity under suboptimal conditions. Bessera et al. [10] reported successful larval development in wastewater, though viability declined markedly (17.2%) in raw sewage. Similarly, Chitolina et al. [11] showed that wastewater, despite slowing larval development and delaying adult emergence by 2–3 days, remained as attractive for oviposition as clean water, with comparable adult emergence rates (*p* = 0.4256).

The physicochemical characteristics of breeding water strongly affect *Aedes'* development. Higher alkalinity reduces larval abundance and adult size, while greater electrical conductivity reflects higher nutrient availability and enhances larval growth [12]. Larval performance depends primarily on the quantity and quality of detritus—especially fresh plant material—rather than container age. Although gravid females prefer older, detritus‐rich containers, these sites may not optimise larval development and can even reduce performance under natural densities because of increased intraspecific competition. Shaded sites and larger container volumes also produce larger adults, potentially increasing vector competence and influencing arbovirus transmission [13].

Complementing the influence of physicochemical parameters, the nutritional landscape of larval habitats constitutes an additional and critical driver of adult vector competence. Nutrient availability in breeding sites—shaped by plant debris and flooding—modulates mosquito vector potential by altering nitrogen (N) and carbon (C) content. Using stable isotope analysis (δ^15^N, δ^13^C), Yee et al. [14] traced these nutrients from larval habitats into adult mosquitoes and demonstrated that individuals with higher N and C levels exhibited greater Zika virus loads. These findings show that larval nutrition directly influences adult vector competence, shaping the ability of mosquitoes to acquire and transmit pathogens.

Urban pollutants markedly influence the biology and life cycle of mosquitoes of the genus *Aedes*. Antonelli et al. [15] showed that polycyclic aromatic hydrocarbons (PAHs), including benzo[a]pyrene and benzo[b]fluoranthene, persist in aquatic habitats for up to 45 days and bioaccumulate in larvae, though not in adults. Larval exposure caused gut dysbiosis, with reduced microbial diversity and increased PAH‐degrading *Comamonadaceae*, while adults exhibited more subtle effects, notably the depletion of key symbionts such as *Wolbachia* and *Cedecea*, potentially impairing immunity, reproduction, and vector competence. Heavy metals (HMs) are likewise present in urban artificial containers. Vargas et al. [16] found that cadmium (Cd) and lead (Pb) accumulate across developmental stages of *Ae. aegypti* and *Ae. albopictus*, whereas copper (Cu) and chromium (Cr) showed negative associations with bioaccumulation, suggesting physiological regulation or tolerance. HM exposure can disrupt mosquito physiology, reproduction, and insecticide resistance, ultimately affecting population dynamics and arbovirus transmission.

Another emerging class of pollutants shown to affect *Aedes* larval survival and vector competence is microplastics (MPs). Griffin et al. [17] reported 37% larval mortality within 48 h at 60 MPs/mL, increasing to 100% at 6000 MPs/mL, with deaths attributed to digestive tract obstruction rather than chemical toxicity. Li et al. [8] further demonstrated that MP exposure reduced Zika virus (ZIKV) vector competence, likely through viral particle adsorption and altered expression of genes involved in immunity and metabolism.

Environmental constraints—such as drought or even temporary scarcity of oviposition sites—may shift female reproductive strategies. Gunathilaka and Ganehiarachchi [18] showed that *Ae. aegypti* females retaining eggs for 8 days laid an average of 100 eggs, a 69% increase over the 59 eggs produced by females ovipositing immediately after blood‐feeding. This difference was significant (*p* < 0.05), with egg output strongly correlated with retention time (R^2^ = 0.986), while fertility, larval mortality, development time, and adult longevity remained unaffected (*p* > 0.05). Thus, egg retention enhances offspring quantity without compromising quality.

Beyond reproductive adjustments, infectious processes also modulate mosquito behaviour. In dengue‐infected *Ae. aegypti*, Javed et al. [19] observed longer flight durations (59.01 s vs. 22.95 s; *p* = 0.0135 × 10^−3^) and greater spatial coverage (*p* = 0.029) despite reduced flight frequency, suggesting infection‐driven increases in host‐seeking persistence. Flight velocity and Euclidean distance did not differ significantly (*p* = 0.064), indicating targeted alterations in flight endurance rather than general locomotion.

Environmental structure also shapes *Aedes* behaviour. Seang‐arwut et al. [20] found peak *Ae. aegypti* abundance at intermediate heights (0.75–1.5 m; *p* < 0.001), matching shaded, sheltered microhabitats near human activity. Indoors, densities were highest in bedrooms (35%–39%; *p* < 0.001) and bathrooms (~30%), reflecting human resting patterns and favourable microhabitat conditions.

Anthropogenic disturbances can also disrupt seasonal regulation. Artificial light at night (ALAN) accelerates larval and pupal development, increases diapause and alters diapause‐related gene expression in *Ae. albopictus* potentially desynchronising life‐cycle timing and promoting urban persistence and geographic expansion [21].

### 3.2 Climatic Variables as Key Drivers of Mosquitoes of the Genus *Aedes* Ecology and Dynamics

Table S3 summarises 46 key findings grouped into three categories: temperature alone, temperature with rainfall, and rainfall as the primary climatic driver of *Aedes* ecology.

Temperature is a key abiotic driver of all *Aedes* life‐cycle stages, strongly influencing abundance, oviposition and larval development [22, 23, 24]. These temperature–abundance relationships create clear seasonality, with activity rising in warmer months and declining in winter [25, 26, 27]. Within this seasonal pattern, urban heat islands raise ambient temperatures further, increasing mosquito fecundity and longevity [24] and strengthening *Aedes* persistence in densely built areas. Species‐specific thermal tolerances shape these responses: *Ae. aegypti* performs best between 21°C and 34°C, can tolerate temperatures up to ~40°C, and reaches peak reproduction and vector competence at 26°C and 30°C [28]. In contrast, *Ae. vexans* and *Ae. geniculatus* peak around 21°C and show marked performance declines above 25°C [29].

Superimposed on these patterns, temperature spikes may act as environmental triggers that precede arboviral outbreaks, accelerating vector and viral development [24, 30]. Additionally, indoor microhabitats being warmer and more stable can accelerate immature development by 22%–37% [31].

Despite warming's general benefits, direct sunlight can reduce egg density and survival, underscoring context‐dependent thermal responses [31, 32]. Given the dependence of mosquito biology, thermal conditions also critically shape arbovirus transmission replication [33, 34]. For instance, ZIKV replication and vector competence have been demonstrated under controlled laboratory conditions around 28°C [35, 36], though DENV‐2 serotype mutations enable efficient replication at suboptimal temperatures (20°C) [37], potentially extending transmission into cooler habitats [38].

Temperature and humidity act synergistically, with entomological indices peaking in warm, humid periods that drive seasonal arthropod dynamics [39, 40, 41, 42, 43, 44, 45, 46, 47]. These variables are widely used to predict *Aedes* populations and anticipate arbovirus outbreaks [43, 48, 49, 50].

While precipitation provides favourable conditions for the proliferation of *Ae. aegypti*, the absence of adequate humidity induces significant physiological and behavioural adaptations in the species. Notably, under arid conditions (30%–40% relative humidity), female *Ae. aegypti* exhibited a marked increase in multiple blood‐feeding behaviour within a single gonotrophic cycle. On average, mosquitoes subjected to low humidity took two to three additional blood meals prior to oviposition—a difference that was statistically significant (*p* < 0.05) compared to cohorts maintained under high‐humidity conditions [51].

The influence of these climatic factors is further amplified within the broader context of climate change which is fundamentally reshaping the ecological niche of Aedes mosquitoes. By persistently altering temperature regimes and rainfall patterns, climate change facilitates the expansion of suitable habitats into new latitudes and elevations, concurrently modifying the dynamics of seasonal transmission. An applied example comes from Réunion Island, where projected warming combined with declining precipitation is expected to suppress *Ae. albopictus* populations in increasingly hot, arid lowlands but enhance suitability at mid‐ and high altitudes, promoting upslope expansion and elevating dengue risk [52]. On a broader geographic scale, projections for the Americas show that both *Ae. aegypti* and *Ae. albopictus* will substantially expand their climatically suitable ranges throughout the 21st century, with strong increases in areas of high habitat suitability, particularly under high‐warming scenarios, driven primarily by rising mean annual temperatures [53]. These warming trends are consistent with global patterns in which climate change pushes *Aedes* distributions poleward, enables colonisation of higher altitudes, and lengthens transmission seasons by sustaining vector activity into late autumn [28, 54].

### 3.3 Urbanisation and Human Practices in the Modulation of Mosquitoes of the Genus *Aedes* Populations

Within the thematic axis Urbanisation and Human Practices in the Modulation of *Aedes* Mosquito Populations, Table S4 synthesises 61 key findings from the reviewed studies across five analytical domains: (1) anthropogenic factors; (2) urban infrastructure and environmental determinants; (3) features of artificial breeding sites; (4) household environment and socioeconomic conditions; and (5) population density and urbanisation as they relate to Aedes distribution and arbovirus transmission.

Socioeconomic vulnerabilities—low income, low education, and poor housing—correlate with higher infestation indices. Greater mosquito vector presence was observed in areas with a higher proportion of households (*p* < 0.01), increased household occupancy (*p* < 0.01), and broader access to water (*p* < 0.05) and sewage networks (*p* < 0.01), regardless of household income. Conversely, dengue cases concentrated in areas characterised by higher household income (*p* < 0.01), a greater proportion of secondary education (*p* < 0.01), lower household occupancy (*p* < 0.01), and improved sewage infrastructure (*p* < 0.01), indicative of a middle‐class profile [55]. Another study corroborated that lower socioeconomic strata had higher vector prevalence, while higher strata had reduced odds of infestation (OR = 0.4). Educational attainment of at least 1 year protected against immature mosquito presence (adjusted OR = 0.3) [56]. Bakhsh et al. [57] demonstrated a positive association between years of schooling and the adoption of dengue prevention measures; each additional year of education increased the adoption of preventive behaviours (coefficient: 0.25, *p* < 0.05). These studies reinforce the central role of educational and household variables in dengue protection.

Spatial analyses show that social inequality, precarious infrastructure, and rapid, unplanned urban expansion create conditions that sustain arbovirus transmission. In Tocantins, Brazil, Gomes et al. [58] found that severe socioeconomic vulnerability and limited basic healthcare coverage facilitate both the spread and persistence of these viruses. Such gradients appear in spatial indicators: dengue incidence increases with population density (*ρ* = 0.31; *p* < 0.05) and decreases with residential area per capita (*ρ* = −0.27; *p* < 0.05), reflecting intensified human–vector contact [59]. Poverty also shapes vector ecology, with higher poverty rates linked to greater *Ae. albopictus* abundance (R^2^ = 0.53; *p* = 0.026). Typical breeding sites in socially degraded areas include waste accumulation zones, mechanical workshops, scrapyards, and domestic water containers [38, 60], and the species' establishment in informal settlements of Rio de Janeiro (Brazil) illustrates this environmental exploitation [61].

Socioeconomic vulnerability and deficient urban infrastructure are strong predictors of dengue incidence, acting primarily through failures in water supply and housing quality. Lack of piped water forces reliance on stored water, increasing habitat availability. This mechanism contributed to elevated infection rates in Sri Lanka (baseline IRR = 1.97, 95% CI: 1.08–3.65; peak August IRR = 6.11, 95% CI: 2.83–13.47) and Thailand (baseline IRR = 14.56, 95% CI: 5.55–39.90; peak June IRR = 23.61, 95% CI: 9.39–61.67). Conversely, improved access to healthcare enhances adaptive capacity, highlighting that infrastructure investments modify vulnerability gradients [62].

Urban infrastructure and socioeconomic conditions strongly influence breeding‐site availability [38, 63]. In Mato Grosso do Sul (Brazil), improved sanitation correlated with stronger rainfall–dengue associations: cities with higher piped water coverage showed strong cross‐correlations with dengue (*ρ* = 0.679; *p* < 0.05), and sewage coverage correlated even more strongly (*ρ* = 0.829; *p* < 0.05) [48]. Infrastructure decay also sustains vector populations; disused irrigation canals in arid San Juan (Argentina) remained productive *Ae. aegypti* sites even under dry conditions [64].

Household water storage is a major determinant of *Aedes* proliferation. In Mexico, piped water access reduces infestation *odd*s by 88% (aOR = 0.12; 95% CI: 0.02–0.73) compared to households relying on stored water. Lack of piped water in Mexico similarly increased *Ae. aegypti* presence (OR = 0.12; 95% CI: 0.02–0.73) [41]. In India, irregular supply was more common in rural areas (21.6% vs. 8.3%; *p* < 0.05), resulting in higher entomological indices (HI, House Index = 22.5%, CI, Container Index = 6.5%, BI, Breteau Index = 40.7%) compared to urban areas (HI = 4.4%, CI = 0.9%, BI = 6.1%) [65]. Across regions, disused, rain‐filled containers—such as tires, buckets, and tanks—are consistently preferred for breeding. In Kenya, 55.2% of immature *Ae. aegypti* were found in rainwater‐filled refuse containers [66], and in India, positivity was significantly higher in rainwater‐filled containers (8.8% vs. 1.9%; *p* < 0.05) and in containers that had never been used (10.7% vs. 0.5%; *p* < 0.05) [65]. Specific species preferences were also observed; for instance, *Ae. albopictus* favours shaded, vegetated areas, whereas *Ae. aegypti* prefers open, sunlit environments and large tires as key container's breeding sites [67, 68]. Moreover, multiple *Aedes* species were found to co‐occur in the same breeding site [68, 69]. Emerging tools, including AI and unmanned aerial vehicles, now allow automated detection of artificial containers—such as tanks and bins—with high predictive performance (pseudo‐R^2^ = 0.74–0.72; F1 ≥ 0.84; *p* ≤ 0.05) [70], despite not seeing inside the houses.

Across different regions, housing conditions and socioeconomic status strongly influence vector proliferation and dengue risk. In Venezuela, Abán Moreyra et al. [71] reported higher egg abundance in wooden houses during February, March, and May (all *p* < 0.05), in very precarious dwellings in January (*p* < 0.05), and a negative correlation for brick houses in April (*p* < 0.05). External water pipes increased egg counts in March (*p* < 0.05), and areas with greater unmet basic needs showed higher egg abundance in February (*p* < 0.05), underscoring the impact of infrastructural deprivation.

Sanitation‐related features also modulate risk. In Cambodia, each additional household toilet corresponded to a 4.36% increase in log‐transformed antibody response (95% CI: 2.56–6.19; *p* < 0.05) [72], suggesting increased human–mosquito exposure even under improved sanitation. In Florida, older housing stock—homes built between 1960 and 1979—harboured significantly higher *Ae. aegypti* densities (both *p* < 0.0001) [23], likely due to outdated water and sanitation systems. In India, the proportion of ‘kuccha’ houses was similar in urban (9.4%) and rural (9.3%) areas (*p* = 0.97), with minimal differences in breeding‐site prevalence [65].

Structural characteristics further influence vector abundance. In rural Thailand, cement‐walled homes (*p* = 0.014), external bathrooms (*p* = 0.017), and a greater number of rooms (*p* = 0.003) were associated with higher mosquito counts [20]. In Shanghai, lower average housing prices—a proxy for deprivation linked to higher *Ae. albopictus* densities in July (coef = −0.653; *p* < 0.001), August (−1.139; *p* < 0.001), and September (−0.709; *p* < 0.01)—Wang et al. [73] findings corroborated by geographically weighted regression. Consistently, Saadatian‐Elahi et al. [74] reported that low‐ and middle‐cost housing, characterised by dense occupation and limited infrastructure, presents greater vulnerability to dengue transmission.

Human and housing density are consistently identified as key determinants of Aedes‐borne diseases. In China, Li et al. [75] observed a significant positive correlation between population density and dengue case counts (Spearman *ρ* = 0.31; *p* < 0.05), indicating that higher human concentration increases vector–host contact. Little et al. [59] likewise found that high‐density housing areas in Puerto Rico were markedly more likely to contain *Ae. aegypti* (OR = 68.99; *p* < 0.001) and co‐occurrence with *Ae. mediovittatus* (OR = 46.15; *p* < 0.001), while continuous urban expanses reduced the latter (OR = 0.13; *p* < 0.001). In Greece, ovitrap monitoring showed significantly greater Ae. albopictus oviposition in the most urbanised subareas (*p* < 0.001) [25]. At the household level in Peru, Padmanabha et al. [76] reported that more occupants per home significantly increased secondary dengue infections (*p* < 0.001) and combined with pupal counts, produced the most predictive epidemic risk index (χ^2^ = 1032.3; log‐likelihood = 62.4). In Hong Kong, spatial dengue risk was similarly driven by urban and environmental factors: the optimal MaxEnt model (AUCTRAIN = 0.847; AUCTEST = 0.841) identified NDVI (31.8%), the frontal area index (22.8%), and aggregation of residential land (17%) as the main predictors of *Ae. albopictus* suitability, reflecting greater favourability in densely populated areas with concentrated housing and moderate vegetation [77]. Complementarily, Pedro et al. [78] developed a vulnerability index incorporating socioeconomic factors such as low income, low education, precarious housing, the presence of children, and suburban characteristics, showing that areas with moderate vegetation, dense built environments and public residential land use were most conducive to mosquito presence.

Land‐use patterns directly shape the presence, abundance and distribution of mosquitoes of the *Aedes* genus, influencing arbovirus transmission [79]. Zahouli et al. [80] documented strong spatial variation in human–mosquito contact: city residents experienced 4.48 bites/day, workers in deforested polyculture areas 21.48, and individuals in forested regions only 0.62. Species responses to landscape context also differ. *Ae. aegypti* remains highly adapted to human environments, whereas *Ae. dendrophilus* is more common in disturbed habitats. Marquetti et al. [81] reported the progressive adaptation of *Ae. albopictus* to Cuban urban areas, noting its growing presence in domestic breeding sites where it accounted for 8%–21.5% (mean 15%) of detections. The species increasingly co‐occurred with *Ae. aegypti*, indicating adjustment to household settings. Consistently, Panda et al. [82] and Câmara et al. [83] found *Ae. aegypti* dominating strictly urban areas, while *Ae. albopictus* occurred broadly across urban and peri‐urban zones.

Therefore, landscape contrasts between urban and rural areas shape mosquito assemblages at ecological transition zones, increasing vector–host interactions and spillover/spillback risk [84]. To support targeted control, Leandro and Maciel [85] developed a georeferenced tool for identifying *Ae. aegypti* hotspots in Brazil, while the GMOD platform provides open‐access, citizen‐generated vector data that enhances surveillance [86]. Furthermore, Rios et al. [87] underscore the critical need for integrated entomological and virological surveillance. Their study documented natural infection of *Ae. aegypti* males with DENV‐4, providing evidence of vertical or venereal transmission as a non‐traditional route of viral spread.

Mathematical models and empirical studies consistently show that human population density is a major driver of *Aedes* ecology and arbovirus transmission. Erguler et al. [50] used population density to scale *Ae. albopictus* carrying capacity, achieving strong agreement with surveillance data (*ρ* = 0.881 in Bologna; *p* < 0.001) despite moderate model sensitivity. Rose et al. [88] found that human density within 20 km explained 18% of the geographic variation in *Ae. aegypti*'s odour‐mediated human preference (likelihood ratio *p* = 1 × 10^−5^). In Texas (USA), low‐density, low‐income neighbourhoods with limited air conditioning and window screens showed higher Zika exposure, whereas high‐density vertical housing reduced mosquito entry [89]. Similarly, in Florida (USA), Uelmen et al. [23] identified human population density and total housing units as highly significant predictors of *Ae. aegypti* abundance (all *p* < 0.0001).

Another study identified a strong correlation between urbanisation and dengue risk, with 3046 geocoded hotspots predominantly clustered in high‐density districts: Petaling (1172; 4755/km^2^), Hulu Langat (828; 1673/km^2^), Klang (414; 1708/km^2^), and Gombak (417; 1452/km^2^). In contrast, low‐density rural areas like Sabak Bernam (4; 107/km^2^) and Kuala Selangor (14; 240/km^2^) reported minimal hotspot activity. These findings underscore high population density and associated urban factors—such as inadequate waste management and abundant artificial water containers—as key drivers of sustained dengue transmission in Selangor [90].

At finer spatial scales, housing structure and microhabitat configuration intensify human‐vector interactions. In Malaysia, Ng et al. [91] showed that high housing density and informal gardens—where pots and ornamental plants accumulate—create focal points for dengue by providing optimal oviposition sites. In China, Khan et al. [92] found that higher human density increases egg recruitment and raises the basic reproduction number (R_0_ > 1 in dense areas), boosting outbreak potential. Lambrechts et al. [35] likewise linked urban density to shifts in *Ae. aegypti*'s human‐specialist lineage, explaining 36% of the variation in Zika seroprevalence (R^2^ = 0.36).

Modelling approaches reinforce these empirical findings. Knoblauch et al. [93] integrated population density within 200‐m buffers around ovitraps into negative‐binomial models that accounted for up to 74% of larval count variability (*p* ≤ 0.05). Regionally, in the Black Sea area, species‐distribution modelling attributed 62.4% of *Ae. albopictus* habitat suitability to human density (Maxent AUC = 0.83) [94]. Conversely, Argibay et al. [60] found that higher human density correlated with lower Chikungunya incidence (RR = 0.78; 95% CI: 0.77–0.80), suggesting that well‐serviced high‐rise areas can limit vector proliferation. Overall, the findings indicate that urban form and socioeconomic context shape breeding sites and transmission, underscoring their relevance for arbovirus control.

Incorporating high‐resolution human mobility data has substantially improved dengue‐transmission forecasting. In Singapore, Massaro et al. [30] showed that an agent‐based model using real commuting patterns from over two million mobile‐phone users achieved the highest temporal prediction accuracy (R^2^ = 0.65), outperforming Lévy (R^2^ = 0.62), radiation (R^2^ = 0.56), and random‐mobility models (R^2^ = 0.51); real‐mobility and radiation models also most closely reproduced observed spatial case patterns according to the Structural Similarity Index (SSIM).

In China, Zhao et al. [95] found that *Ae. albopictus* from port, urban, and rural sites displayed uniformly high genetic diversity (haplotype diversity Hd = 0.8069–0.9678) and low nucleotide diversity—patterns consistent with recent expansion—along with strong, bidirectional gene flow among all locations, particularly between ports and other regions (Nm > 13 in key urban locales). Therefore, intense human‐mediated dispersal prevents population differentiation and hinders vector control. Complementing these patterns, Tu et al. [96] showed that most imported dengue cases from Southeast Asia clustered in districts with major transport hubs, which later experienced the most severe 2019 outbreaks—demonstrating how human mobility drives viral introduction and local transmission.

New repellent‐based technologies increasingly account for urban structures, residential features, and *Aedes* behavioural ecology [97]. In urban New Jersey (USA), nighttime applications—despite *Ae. albopictus* being diurnal—significantly reduced adult populations. Cage trials showed higher mortality in front yards (78%) than backyards (58%; *p* < 0.001) [98]. Environmental chemical control is increasingly limited by growing insecticide resistance. In agroecosystems of Benin, *Ae. aegypti* represented 93.9% of captures and exhibited significant permethrin resistance in two of three areas (*p* = 0.02), with multiple *kdr* mutations and overexpression of P450 genes [99]. In Posadas, Argentina, *kdr* mutations V1016I and F1534C were markedly more frequent in higher‐income neighbourhoods (67% vs. 34% in socioeconomically vulnerable areas), and *Ae. aegypti* abundance averaged 3.32 versus 2.16 mosquitoes per household (*p* < 0.01), suggesting that intensified insecticide use in wealthier districts may inadvertently select for resistant vectors [100].

Larval control and personal‐protection measures show variable effectiveness. Seang‐arwut et al. [20] found that applying temephos every 3 months was inadequate, with low‐frequency use correlating with increased *Ae. aegypti* presence (*p* = 0.024). In urban areas, repellent use was paradoxically associated with higher mosquito counts (*p* = 0.022). By contrast, transfluthrin‐impregnated polyester (TFT‐P) showed strong protection: morning landings decreased by 60.73% (vs. 31.97% in the afternoon; *p* < 0.001), blood‐feeding dropped to 37.5% from 67.7% (*p* = 0.004), and egg hatching to 31.2% from 54.5% (*p* = 0.039), though clutch size was unchanged. Temperature and humidity significantly affected landing rates (*p* = 0.009; *p* = 0.019), underscoring the need to tailor deployment to environmental conditions [101]. Furthermore, Ruggerio et al. [102] identified a crucial challenge in Buenos Aires, Argentina: a pervasive public unawareness of the vector's life cycle and the risks of water containers, which undermined municipal prevention efforts. They conclude that effective risk mitigation necessitates sustained, integrated interventions and enhanced educational campaigns specifically targeting domestic habits.

In Rwanda, Rusanganwa et al. [103] showed that farmers have a markedly higher risk of past dengue infection due to frequent outdoor exposure, whereas office workers (OR = 0.53; 95% CI: 0.32–0.86) and individuals in other occupations (OR = 0.52; 95% CI: 0.34–0.80) had significantly lower odds of DENV IgG seropositivity (*p* < 0.05). Complementing this, Tu et al. [96] found significant differences between imported and locally acquired infections in 2019 with respect to occupational status, gender and age (*p* < 0.05), demonstrating how occupational, demographic, and mobility factors jointly shape dengue transmission dynamics.

### 3.4 One Health Framework in the Study of Aedes‐Borne Arboviruses

Applying the One Health concept through an integrative methodology, 12 of the 98 articles met the inclusion criteria by jointly analysing entomological, climatic–socioenvironmental, and human case data on Aedes‐borne arboviruses. Foundational Brazilian studies [55, 69] demonstrated that arbovirus emergence results from the interplay between human behaviour, environmental modification, and vector ecology—showing that transmission cannot be understood or controlled through biomedical measures alone.

Building on this foundation, recent research has expanded the global relevance of One Health approaches. In Benin, agricultural practices were directly linked to vector ecology and increased arbovirus risk [98]. In China, studies integrating entomological, epidemiological, climatic, environmental, and vector‐density variables with dengue notifications enhanced eco‐epidemiological understanding of transmission [27, 79], while another examined how vector genetics and urban environments shape disease risk [95]. In Brazil, Pedro et al. [78] developed an environmental receptivity index for *Ae. aegypti* based on multidimensional indicators. Collectively, these works show how human activities, ecosystem alteration, and mosquito behavioural responses—such as insecticide resistance, genetic diversity, and density variation—create complex feedback systems central to One Health analyses.

This integrative perspective is reinforced in Malaysia, where Abdullah et al. [49] demonstrated that temperature, humidity, and rainfall influence Aedes density, breeding dynamics, and infection rates, underscoring the value of combining climate and entomological indicators to inform multisectoral, One Health‐oriented surveillance and prevention. Community engagement and institutional action in Argentina further highlighted the power of integrated approaches, successfully reducing *Aedes* populations even under climatically favourable conditions [102]. In Brazil, climatic variability and socioenvironmental factors were associated with Chikungunya incidence [60], and socially vulnerable areas—characterised by poor sanitation and irregular urbanisation, conditions worsened after rainfall—were identified as the most susceptible to *Ae. aegypti* proliferation and dengue outbreaks [48]. Borges et al. [47] likewise conceptualised dengue as an eco‐epidemiological and social phenomenon shaped by climate, urban structures, viral biology and institutional capacity. Together, these studies highlight that effective arbovirus prevention requires a One Health approach that integrates human health, ecological dynamics and social determinants into a unified analytical and operational framework.

## 4 Discussion

The results of this review show that mosquitoes of the genus *Aede*s possess a high degree of adaptive capacity, enabling them to persist across a wide range of environmental and anthropogenic conditions. Across the included studies, the determinants of *Aedes* dynamics clustered into three main domains—climatic, environmental/urban, and socio‐ecological—which frequently interact at the community interface. Overall, temperature and rainfall acted as upstream climatic drivers, while urban structure shaped breeding opportunities and socio‐ecological vulnerability influenced exposure and prevention capacity.

### 4.1 Climatic Determinants: Temperature and Rainfall as Primary Drivers

Among climatic variables, temperature and rainfall were the most consistently reported drivers of population dynamics in mosquitoes of the genus *Aedes*. In multiple settings, vector abundance and entomological indices increased during warmer and wetter seasons, reflecting the combined influence of accelerated mosquito development, improved survival and greater availability of breeding habitats during rainy periods [31, 39, 40, 43, 71]. These findings reinforce the role of climate as a foundational determinant of seasonal fluctuations, particularly in regions where mosquito reproduction remains closely dependent on natural or semi‐natural water availability [31, 39, 40].

### 4.2 Climate Change and Expansion Into Previously Unsuitable Regions

Beyond seasonal variability, climate change emerged as another critical driver shaping the dynamics of mosquito populations of the genus *Aedes*, particularly through its influence on geographic expansion. Rising global temperatures have facilitated the spread of *Aedes* mosquitoes into areas previously considered unsuitable, altering ecological boundaries and increasing the risk of arbovirus emergence in new contexts [29, 54]. This expansion has important public health implications, including the potential circulation of established arboviruses and heightened concern regarding spillover and emergence events involving pathogens such as Mayaro and Venezuelan equine encephalitis viruses [104, 105]. In newly colonised regions, the interaction between temperature and water availability appears especially relevant, with studies reporting elevated entomological indices when these factors converge [42]. Thus, the findings indicate that *Aedes* species are not only highly successful in consolidated urban environments but are also gaining new ecological territory due to ongoing climatic shifts.

### 4.3 Environmental and Urban Determinants: Artificial Breeding Sites and Microclimatic Enhancement

Although climatic drivers were prominent, the findings of this review also indicate that, in densely urbanised or arid environments, mosquito populations of the genus *Aedes* may become substantially less dependent on climatic variability. In these contexts, the most influential environmental determinant is the availability of artificial breeding sites, especially water stored in containers, which provides stable oviposition sites regardless of rainfall patterns [47, 68, 94]. This mechanism is particularly relevant in areas with irregular water supply or limited infrastructure, where household storage practices sustain mosquito reproduction even during dry seasons [47, 68]. In addition, several studies highlighted that urban microclimatic conditions—such as heat island effects and artificial night lighting—may contribute to mosquito longevity and survival, thereby increasing opportunities for arbovirus transmission [21, 24, 31]. Together, these results suggest that urbanisation not only increases breeding opportunities through artificial habitats but may also enhance vector persistence through microclimatic modulation.

### 4.4 Socio‐Ecological Determinants: Infrastructure, Vulnerability, and Household‐Level Patterns

Socio‐ecological determinants were also central to infestation patterns, particularly where environmental exposure is compounded by structural vulnerability. Across studies, inadequate infrastructure was repeatedly associated with conditions that promote artificial breeding sites, largely linked to household water storage and accumulated waste [41, 65]. Social and educational vulnerabilities may further limit the adoption of preventive behaviours and sustained community engagement in vector control [66], while unequal access to health services shapes gradients of risk and adaptive capacity across communities [62]. At the household scale, housing characteristics contributed to spatial heterogeneity: wooden structures were associated with higher egg counts [71], and the number of bathrooms was identified as a strong predictor of indoor infestation [23]. These associations align with the resting behaviour of *Aedes* mosquitoes, which tend to occupy shaded, mid‐level indoor microhabitats—particularly bedrooms and bathrooms—where human presence and favourable environmental conditions coincide [20]. Although many studies focused on *Ae. aegypti* and *Ae. albopictus*, the mechanisms synthesised in this review reflect broader adaptive patterns across the genus *Aedes*, supporting their ecological success in human‐modified environments.

### 4.5 Implications for Vector Control: Operational Limits and Behavioural Plasticity

The findings of this review have direct implications for vector control, particularly in urban settings where traditional strategies may face operational constraints. While source reduction remains a cornerstone of mosquito control programs in Brazil, its impact is often limited in densely built environments where recolonisation may occur rapidly and where breeding sites are continuously replenished by household practices and urban infrastructure conditions [106]. Behavioural plasticity further intensifies this challenge: in the absence of suitable oviposition sites, females can retain eggs for up to eight days without compromising offspring viability [18], enabling swift population recovery once conditions become favourable. These findings support the need for interventions that integrate behavioural ecology with the physical realities of urban design, emphasising both structural solutions (e.g., improved water supply and waste management) and sustained community‐level prevention.

### 4.6 One Health Perspective: Integrated Determinants at the Community Interface

Collectively, these dynamics underscore the relevance of a One Health perspective. This framework recognises the interdependence between human vulnerability, environmental structure and vector biology, allowing a more comprehensive interpretation of arbovirus emergence and persistence at the community interface. From a One Health standpoint, most emerging infectious diseases originate in animals and are intensified by human‐driven processes such as urban expansion and ecosystem disruption [107]. Accordingly, the evidence synthesised in this review points to the need for cross‐sector and multidisciplinary interventions that integrate surveillance, vector control, urban planning, and social policy, particularly in settings where climatic suitability converges with socio‐ecological vulnerability. These findings are supported by recent reviews [108, 109], which argue that vector control is a key component of arboviral disease prevention and requires robust governance structures, meaningful community engagement, and intersectoral coordination, rather than isolated, short‐term campaigns. Therefore, integrated vector management is closely aligned with One Health principles, as it strengthens surveillance, enables coordinated responses, reduces ecological risks, addresses insecticide resistance and improves community acceptance.

### 4.7 Limitations and Future Directions

This review also presents limitations. Regional and interspecific heterogeneity was substantial, as different Aedes species display distinct breeding‐site preferences, environmental tolerances and behavioural traits, which may reduce generalisability across contexts. Methodological variability—driven largely by ecological and epidemiological study designs—and the potential for publication bias further constrain comparability between studies. These limitations highlight the importance of standardised methodologies, harmonised entomological metrics, and surveillance systems tailored to local contexts, which are needed to improve predictive accuracy and guide more robust, context‐sensitive vector‐control strategies.

## Funding

Grand Challenges—Impact of Climate Change on Health – Bill & Melinda Gates Foundation and CNPq Grant 407746/2024‐2 – A One Health Approach to Modeling Aedes‐Transmitted Arboviruses in Brazil

## Conflicts of Interest

The authors declare no conflicts of interest.

## Supporting information


**Supporting Information S1:** Full Search Strategy and Search Log.

**Table S2:** Influence of Water Quality and Environmental Factors on Mosquitoes of the Genus *Aedes*.

**Table S3:** Influence of Temperature on the Biology, Ecology, and Vector Potential of Mosquitoes of the Genus *Aedes*.

**Table S4:** Anthropogenic Factors Influencing the Reproduction, Viability, and Persistence of Mosquitoes of the Genus *Aedes*.

## Data Availability

The data that supports the findings of this study are available in the Supporting Information of this article.

## References

[1] E. Abbasi , “Global Expansion of Aedes Mosquitoes and Their Role in the Transboundary Spread of Emerging Arboviral Diseases: A Comprehensive Review,” IJID One Health 6 (2025): 100058, 10.1016/j.ijidoh.2025.100058.

[2] The Lancet , “Dengue: The Threat to Health Now and in the Future,” Lancet 404, no. 10450 (2024): 311, 10.1016/S0140-6736(24)01542-3. 39067890

[3] M. U. G. Kraemer , R. C. Reiner , O. J. Brady , et al., “Past and Future Spread of the Arbovirus Vectors *Aedes aegypti* and *Aedes albopictus* ,” Nature Microbiology 4, no. 5 (2019): 854–863, 10.1038/s41564-019-0376-y. PMC6522366 30833735

[4] A. S. Leandro , R. D. Lopes , C. A. Martins , et al., “The Adoption of the One Health Approach to Improve Surveillance of Venomous Animal Injury, Vector‐Borne and Zoonotic Diseases in Foz Do Iguaçu, Brazil,” PLoS Neglected Tropical Diseases 15, no. 2 (2021): e0009109, 10.1371/journal.pntd.0009109. 33600424 PMC7891772

[5] M. J. Page , J. E. McKenzie , P. M. Bossuyt , et al., “The PRISMA 2020 Statement: An Updated Guideline for Reporting Systematic Reviews,” BMJ (Clinical Research Edition) (2021): n71, v.372 10.1136/bmj.n71. PMC8005924 33782057

[6] Zotero , Version 6.0.26 [Computer Software] (Corporation for Digital Scholarship, 2023), https://www.zotero.org.

[7] Microsoft Corporation , Microsoft Excel [Computer Program] (Microsoft Corporation, 2026).

[8] J. Li , X. Liu , H. Gao , G. Liang , T. Zhao , and C. Li , “Not for Nothing, Microplastics Can (Potentially) Reduce the Risk of Mosquito‐To‐Human Transmission of Arboviruses,” Journal of Hazardous Materials 492 (2025): 138166, 10.1016/j.jhazmat.2025.138166. 40209412

[9] Lubna , Shabana Bibi B. Rasheed , and Farah Zaidi , “Species Diversity Pattern of Mosquitoes (Diptera: Culicidae) Breeding in Different Permanent, Temporary and Natural Container Habitats of Peshawar, KP Pakistan,” Brazilian Journal of Biology 84 (2023) 1–15, 10.1590/1519-6984.271524. 37194758

[10] E. B. Beserra , E. M. de Freitas , J. T. de Souza , C. R. M. Fernandes , and K. D. Santos , “Ciclo de Vida de *Aedes (Stegomyia) aegypti* (Diptera, Culicidae) em Águas Com Diferentes Características,” Iheringia. Série Zoologia 99, no. 3 (2009): 281–285, 10.1590/S0073-47212009000300007.

[11] R. F. Chitolina , F. A. Anjos , T. S. Lima , E. A. Castro , and M. C. V. Costa‐Ribeiro , “Raw Sewage as Breeding Site to *Aedes* (*Stegomyia*) *Aegypti* (Diptera, Culicidae),” Acta Tropica 164 (2016): 290–296, 10.1016/j.actatropica.2016.07.013. 27640323

[12] W. M. Ouédraogo , K. H. Toé , A. Sombié , et al., “Impact of Physicochemical Parameters of *Aedes aegypti* Breeding Habitats on Mosquito Productivity and the Size of Emerged Adult Mosquitoes in Ouagadougou City, Burkina Faso,” Parasites & Vectors 15, no. 1 (2022): 478, 10.1186/s13071-022-05558-3. 36539816 PMC9768987

[13] P. Montini and S. Fischer , “Oviposition Site Selection and Subsequent Offspring Performance of *Aedes aegypti* in Short‐ and Long‐Term Detritus Accumulation Conditions,” Acta Tropica 255 (2024): 107222, 10.1016/j.actatropica.2024.107222. 38685339

[14] S. H. Yee , D. A. Yee , R. de Jesus Crespo , A. Oczkowski , F. Bai , and S. Friedman , “Linking Water Quality to *Aedes aegypti* and Zika in Flood‐Prone Neighborhoods,” EcoHealth 16, no. 2 (2019): 191–209, 10.1007/s10393-019-01406-6. 30945160 PMC7163161

[15] P. Antonelli , S. Grizard , F. H. Tran , et al., “Bioaccumulation of Polycyclic Aromatic Hydrocarbons and Microbiota Dynamics Across Developmental Stages of the Asian Tiger Mosquito, *Aedes albopictus* Exposed to Urban Pollutants,” Ecotoxicology and Environmental Safety 286 (2024): 117214, 10.1016/j.ecoenv.2024.117214. 39447296

[16] V. Vargas , R. García‐Martínez , K. E. Nava‐Castro , et al., “Detection of Heavy Metals in Various Stages of Development for Wild Mosquitoes of *Aedes aegypti* and *Aedes albopictus* Sourced From Artificial Aquatic Niches in Arbovirus Endemic Areas,” Science of the Total Environment 981 (2025): 179551, 10.1016/j.scitotenv.2025.179551. 40347752

[17] C. D. Griffin , C. Tominiko , M. C. I. Medeiros , and J. W. Walguarnery , “Microplastic Pollution Differentially Affects Development of Disease‐Vectoring *Aedes* and *Culex* Mosquitoes,” Ecotoxicology and Environmental Safety 267 (2023): 115639, 10.1016/j.ecoenv.2023.115639. 37924798

[18] R. A. K. M. Gunathilaka and G. A. S. M. Ganehiarachchi , “Effect of Oviposition‐Site Deprivation on Reproductive Performance and Life History Parameters of Dengue Vector *Aedes aegypti* ,” Journal of the National Science Foundation of Sri Lanka 51, no. 1 (2023): 3–11, 10.4038/jnsfsr.v51i1.10770. 36065760

[19] N. Javed , A. J. López‐Denman , P. N. Paradkar , and A. Bhatti , “Flight Traits of Dengue‐Infected *Aedes aegypti* Mosquitoes,” Computers in Biology and Medicine 171 (2024): 108178, 10.1016/j.compbiomed.2024.108178. 38394802

[20] C. Seang‐arwut , Y. Hanboonsong , V. Muenworn , et al., “Indoor Resting Behavior of *Aedes aegypti* (Diptera: Culicidae) in Northeastern Thailand,” Parasites & Vectors 16, no. 1 (2023): 127, 10.1186/s13071-023-05746-9. 37060087 PMC10103527

[21] Q. Liu , H. D. Zhang , D. Xing , et al., “The Effect of Artificial Light at Night (ALAN) on the Characteristics of Diapause of Aedes Albopictus,” Science of the Total Environment 924 (2024): 171594, 10.1016/j.scitotenv.2024.171594. 38461989

[22] O. J. Brady , M. A. Johansson , C. A. Guerra , et al., “Modelling Adult *Aedes aegypti* and *Aedes albopictus* Survival at Different Temperatures in Laboratory and Field Settings,” Parasites & Vectors 6, no. 1 (2013): 351, http://www.parasitesandvectors.com/content/6/1/351. 24330720 10.1186/1756-3305-6-351 PMC3867219

[23] J. A. Uelmen , C. D. Mapes , A. Prasauskas , et al., “A Habitat Model for Disease Vector *Aedes aegypti* in the Tampa Bay Area, Florida,” Journal of the American Mosquito Control Association 39, no. 2 (2023): 96–107, 10.2987/23-7210. 37364184

[24] D. Novianto , U. K. Hadi , S. Soviana , and H. S. Darusman , “Comparison of Diurnal Biting Activity, Life Table, and Demographic Attributes of *Aedes albopictus* (Asian Tiger Mosquito) From Different Urbanized Settings in West Java, Indonesia,” Acta Tropica 241 (2023): 106771, 10.1016/j.actatropica.2022.106771. 36414048

[25] A. Giatropoulos , N. Emmanouel , G. Koliopoulos , and A. Michaelakis , “A Study on Distribution and Seasonal Abundance of *Aedes albopictus* (Diptera: Culicidae) Population in Athens, Greece,” Journal of Medical Entomology 49, no. 2 (2012): 262–269, 10.1603/me11096. 22493842

[26] E. Abbasi , “The Impact of Climate Change on *Aedes aegypti* Distribution and Dengue Fever Prevalence in Semi‐Arid Regions: A Case Study of Tehran Province, Iran,” Environmental Research 275 (2025): 121441, 10.1016/j.envres.2025.121441. 40118318

[27] Y. Zhou , H. Liu , P. Leng , et al., “Analysis of the Spatial Distribution of *Aedes albopictus* in an Urban Area of Shanghai, China,” Parasites & Vectors 14, no. 1 (2021): 501, 10.1186/s13071-021-05022-8. 34565466 PMC8474869

[28] M. Kamal , M. A. Kenawy , M. H. Rady , A. S. Khaled , and A. M. Samy , “Mapping the Global Potential Distributions of Two Arboviral Vectors *Aedes aegypti* and *ae. Albopictus* Under Changing Climate,” PLoS One 13, no. 12 (2018): e0210122, 10.1371/journal.pone.0210122. 30596764 PMC6312308

[29] S. H. Nikookar , A. Charkame , A. Nezammahalleh , et al., “Entomological Surveillance of Invasive *Aedes* Mosquitoes in Mazandaran Province, Northern Iran From 2014 to 2020,” Scientific Reports 13, no. 1 (2023): 35860, 10.1038/s41598-023-35860-8. PMC10227060 37248286

[30] E. Massaro , D. Kondor , and C. Ratti , “Assessing the Interplay Between Human Mobility and Mosquito Borne Diseases in Urban Environments,” Scientific Reports 9, no. 1 (2019): 53127, 10.1038/s41598-019-53127-z. PMC6858332 31729435

[31] G. Cui , S. Zhong , T. Zheng , et al., “ *Aedes albopictus* Life Table: Environment, Food, and Age Dependence Survivorship and Reproduction in a Tropical Area,” Parasites & Vectors 14, no. 1 (2021): 508, 10.1186/s13071-021-05081-x. 34743753 PMC8573987

[32] P. S. Musunzaji , B. A. Ndenga , S. Mzee , et al., “Oviposition Preferences of *Aedes aegypti* in Msambweni, Kwale County, Kenya,” Journal of the American Mosquito Control Association 39, no. 2 (2023): 85–95, 10.2987/22-7103. 37270926 PMC10885850

[33] S. Cunze , J. Kochmann , L. K. Koch , K. J. Hasselmann , and S. Klimpel , “Vector Distribution and Transmission Risk of the Zika Virus in South and Central America,” PeerJ 7 (2019): e7920, 10.7717/peerj.7920. 31745446 PMC6863140

[34] M. C. Wimberly , J. K. Davis , M. V. Evans , et al., “Land Cover Affects Microclimate and Temperature Suitability for Arbovirus Transmission in an Urban Landscape,” PLoS Neglected Tropical Diseases 14, no. 9 (2020): e0008614, 10.1371/journal.pntd.0008614. 32956355 PMC7529312

[35] L. Lambrechts , J. M. Caldwell , and N. H. Rose , “The Role of Vector Population Variation and Climate in Zika Virus Transmission Patterns in Africa: A Modelling Study,” Lancet Planetary Health 8, no. 12 (2024): e1020–e1029, https://www.thelancet.com/journals/lanplh/article/PIIS2542‐5196(24)00276‐6/fulltext. 39674192 10.1016/S2542-5196(24)00276-6 PMC12352338

[36] J. R. Weger‐Lucarelli , A. Ruckert , A. Chotiwan , et al., “Vector Competence of American Mosquitoes for Three Strains of Zika Virus,” PLoS Neglected Tropical Diseases 10, no. 10 (2016): e0005101, 10.1371/journal.pntd.0005101. 27783679 PMC5081193

[37] H. Y. Ko , Y. T. Li , H. P. Yu , et al., “Emergence and Increased Epidemic Potential of Dengue Variants With the NS5V357E Mutation After Consecutive Years of Transmission,” iScience 27, no. 11 (2024): 110899, 10.1016/j.isci.2024.110899. 39524326 PMC11550591

[38] S. Knoblauch , R. T. Mukaratirwa , P. F. P. Pimenta , et al., “Urban *Aedes aegypti* Suitability Indicators: A Study in Rio de Janeiro, Brazil,” Lancet Planetary Health 9, no. 4 (2025): e264–e273, 10.1016/S2542-5196(25)00049-X. 40252673

[39] P. L. Neto and M. A. Navarro‐Silva , “Development, Longevity, Gonotrophic Cycle and Oviposition of *Aedes albopictus* Skuse (Diptera: Culicidae) Under Cyclic Temperatures,” Neotropical Entomology 33, no. 1 (2004): 29–33, 10.1590/S1519-566X2004000100005.

[40] M. Stein , G. I. Oria , W. R. Almirón , and J. A. Willener , “Seasonal Fluctuation of *Aedes aegypti* in Chaco Province, Argentina,” Revista de Saúde Pública 39, no. 4 (2005): 559–564, 10.1590/S0034-89102005000400007. 16113904

[41] M. H. Hayden , C. K. Uejio , K. Walker , et al., “Microclimate and Human Factors in the Divergent Ecology of *Aedes aegypti* Along the Arizona, U.S./Sonora, MX Border,” EcoHealth 7, no. 1 (2010): 64–77, 10.1007/s10393-010-0288-9. 20232228

[42] J. A. Rader , A. Serrato‐Capuchina , T. Anspach , and D. R. Matute , “The Spread of *Aedes albopictus* (Diptera: Culicidae) in the Islands of São Tomé and Príncipe,” Acta Tropica 251 (2024): 107106, 10.1016/j.actatropica.2023.107106. 38185188 PMC11559242

[43] I. V. G. Borges , A. Musah , L. M. M. Dutra , et al., “Analysis of the Interrelationship Between Precipitation and Confirmed Dengue Cases in the City of Recife (Brazil) Covering Climate and Public Health Information,” Frontiers in Public Health 12 (2024): 1456043, 10.3389/fpubh.2024.1456043. 39507663 PMC11537940

[44] Z. Farooq , L. Segelmark , J. Rocklöv , et al., “Impact of Climate and *Aedes albopictus* Establishment on Dengue and Chikungunya Outbreaks in Europe: A Time‐To‐Event Analysis,” Lancet Planetary Health 9, no. 5 (2025): e374–e383, 10.1016/S2542-5196(25)00059-2. 40381632

[45] M. A. O. Nasif , N. Haider , I. Muntasir , et al., “The Reappearance of Chikungunya Virus in Bangladesh, 2024,” IJID Regions 16 (2025): 100664, 10.1016/j.ijregi.2025.100664. 40606592 PMC12209986

[46] X. Wang , S. Tang , J. Wu , Y. Xiao , and R. A. Cheke , “A Combination of Climatic Conditions Determines Major Within‐Season Dengue Outbreaks in Guangdong Province, China,” Parasites & Vectors 12, no. 1 (2019): 45, 10.1186/s13071-019-3295-0. 30665469 PMC6341621

[47] H. Qureshi , M. I. Khan , S. J. Bae , et al., “Prevalence of Dengue Virus in Haripur District, Khyber Pakhtunkhwa, Pakistan,” Journal of Infection and Public Health 16, no. 7 (2023): 1131–1136, 10.1016/j.jiph.2023.04.021. 37244095

[48] J. B. Oliveira , T. B. Murari , A. S. Nascimento Filho , H. Saba , M. A. Moret , and C. A. L. Cardoso , “Paradox Between Adequate Sanitation and Rainfall in Dengue Fever Cases,” Science of the Total Environment 860 (2023): 160491, 10.1016/j.scitotenv.2022.160491. 36455745

[49] N. A. M. H. Abdullah , N. C. Dom , B. Pradhan , S. A. Salleh , and R. Dapari , “Temporal Associations Between Microclimate, Adult *Aedes* Mosquito Indices, and Dengue Cases at the Residence Level in Malaysia: Implications for Targeted Interventions,” PLoS One 20, no. 2 (2025): e0316564, 10.1371/journal.pone.0316564. 39899560 PMC11790129

[50] K. Erguler , S. E. Smith‐Unna , J. Waldock , et al., “Large‐Scale Modelling of the Environmentally‐Driven Population Dynamics of Temperate *Aedes albopictus* (Skuse),” PLoS One 11, no. 2 (2016): e0149282, 10.1371/journal.pone.0149282. 26871447 PMC4752251

[51] C. J. Holmes , S. Chakraborty , O. M. Ajayi , et al., “Multiple Blood Feeding Bouts in Mosquitoes Allow for Prolonged Survival and Are Predicted to Increase Viral Transmission During Dry Periods,” iScience 28, no. 2 (2025): 111760, 10.1016/j.isci.2025.111760. 39935457 PMC11810705

[52] K. Lamy , A. Tran , T. Portafaix , M. D. Leroux , and T. Baldet , “Impact of Regional Climate Change on the Mosquito Vector *Aedes albopictus* in a Tropical Island Environment: La Réunion,” Science of the Total Environment 875 (2023): 162484, 10.1016/j.scitotenv.2023.162484. 36889019

[53] M. E. Gorris , L. Espinosa , J. Yoo , S. Cavany , C. M. Barker , and R. J. Eisen , “Climate and Land‐Use Change Drive Future Shifts in Mosquito Habitat Suitability Across the Americas,” Science of the Total Environment 923 (2024): 171836, 10.1016/j.scitotenv.2024.171836.

[54] A. R. Kaye , U. Obolski , L. Sun , et al., “The Impact of Natural Climate Variability on the Global Distribution of *Aedes aegypti* : A Mathematical Modelling Study,” Lancet Planetary Health 8, no. 12 (2024): e1079–e1087, 10.1016/S2542-5196(24)00238-9. 39674197 PMC7617884

[55] C. Barcellos , A. Kreutz Pustai , M. A. Weber , M. Regina , and V. Brito , “Identificação de locais com potencial de transmissão de dengue em Porto Alegre através de técnicas de geoprocessamento,” Revista da Sociedade Brasileira de Medicina Tropical 38, no. 3 (2005): 246–250, 10.1590/s0037-86822005000300007. 15895177

[56] J. Quintero , G. Carrasquilla , R. Suárez , C. González , and V. A. Olano , “An Ecosystemic Approach to Evaluating Ecological, Socioeconomic and Group Dynamics Affecting the Prevalence of *Aedes aegypti* in Two Colombian Towns,” Cadernos de Saúde Pública 25, no. suppl. 1 (2009): S93–S103, 10.1590/s0102-311x2009001300009. 19287871

[57] K. Bakhsh , F. Sana , and N. Ahmad , “Dengue Fever in Punjab, Pakistan: Knowledge, Perception and Adaptation Among Urban Adults,” Science of the Total Environment 644 (2018): 1304–1311, 10.1016/j.scitotenv.2018.07.077. 30743843

[58] H. Gomes , A. G. de Jesus , and J. A. S. Quaresma , “Identification of Risk Areas for Arboviruses Transmitted by *Aedes aegypti* in Northern Brazil: A One Health Analysis,” One Health 16 (2023): 100499, 10.1016/j.onehlt.2023.100499. 36844974 PMC9945760

[59] E. Little , R. Barrera , K. C. Seto , and M. Diuk‐Wasser , “Co‐Occurrence Patterns of the Dengue Vector Aedes Aegypti and Aedes Mediovittatus, a Dengue‐Competent Mosquito in Puerto Rico,” EcoHealth 8, no. 3 (2011): 365–375, 10.1007/s10393-011-0708-8. 21989642 PMC4646052

[60] H. D. Argibay , C. W. Cardoso , W. M. de Souza , et al., “High‐Resolution Spatiotemporal Analysis of Chikungunya Epidemics Between 2019 and 2020 in Salvador, Brazil: A Municipality‐Level Transmission Dynamics Study,” Lancet Regional Health ‐ Americas 43 (2025): 101003, 10.1016/j.lana.2025.101003. 39925861 PMC11804771

[61] T. Ayllón , D. C. P. Câmara , F. C. Morone , et al., “Dispersion and Oviposition of *Aedes albopictus* in a Brazilian Slum: Initial Evidence of Asian Tiger Mosquito Domiciliation in Urban Environments,” PLoS One 13, no. 4 (2018): e0195014, 10.1371/journal.pone.0195014. 29684029 PMC5912725

[62] Y. Wang , C. Li , S. Zhao , et al., “Evaluation of Dengue Fever Vulnerability in South and Southeast Asian Countries: A Multidimensional Approach,” Journal of Infection and Public Health 18, no. 9 (2025): 102849, 10.1016/j.jiph.2025.102849. 40472480

[63] L. D. Ortega‐López , M. P. Betancourth , R. León , A. Kohl , and H. M. Ferguson , “Behavior and Distribution of *Aedes aegypti* Mosquitoes and Their Relation to Dengue Incidence in Two Transmission Hotspots in Coastal Ecuador,” PLoS Neglected Tropical Diseases 18, no. 4 (2024): e0010932, 10.1371/journal.pntd.0010932. 38683840 PMC11081501

[64] E. Illa , F. Murúa , F. H. Aballay , et al., “ *Aedes* (*Stegomyia*) *Aegypti* in Ditches From an Arid Region of Argentina,” Journal of Arid Environments 223 (2024): 105194, 10.1016/j.jaridenv.2024.105194.

[65] G. Singh , R. Tilak , and S. K. Kaushik , “Bio‐Eco‐Social Determinants of *Aedes* Breeding in Field Practice Area of a Medical College in Pune, Maharashtra,” Indian Journal of Public Health 63, no. 4 (2019): 324–329, 10.4103/ijph.IJPH_296_18. 32189652

[66] J. E. Forsyth , F. M. Mutuku , L. Kibe , et al., “Source Reduction With a Purpose: Mosquito Ecology and Community Perspectives Offer Insights for Improving Household Mosquito Management in Coastal Kenya,” PLoS Neglected Tropical Diseases 14, no. 5 (2020): e0008239, 10.1371/journal.pntd.0008239. 32392226 PMC7241847

[67] J. A. Achaga and D. Vezzani , “A Methodological Proposal to Estimate the Total Abundance of Immature Mosquitoes in Discarded Tyres: Aedes Aegypti and *Culex pipiens* as Study Cases,” Acta Tropica 260 (2024): 107474, 10.1016/j.actatropica.2024.107474. 39551419

[68] A. S. Babalola , A. O. Adeogun , H. S. Thabet , et al., “Geospatial Modeling of Geographical Spread of *Aedes* Species, in Relation to Climatic and Topographical Factors in Lagos State, Nigeria,” PLoS Neglected Tropical Diseases 19, no. 2 (2025): e0012860, 10.1371/journal.pntd.0012860. 39913605 PMC11825102

[69] J. A. C. Zequi , J. Lopes , and I. M. Medri , “Imaturos de *Culicidae* (*Diptera*) encontrados em recipientes instalados em mata residual no município de Londrina, Paraná, Brasil,” Revista Brasileira de Zoologia 22, no. 3 (2005): 656–661, 10.1590/S0101-81752005000300015.

[70] K. Yu , J. Wu , M. Wang , et al., “Using UAV Images and Deep Learning in Investigating Potential Breeding Sites of *Aedes albopictus* ,” Acta Tropica 255 (2024): 107234, 10.1016/j.actatropica.2024.107234. 38688444

[71] D. N. Abán Moreyra , P. M. Castillo , A. Escalada , et al., “Use of *Aedes aegypti* Oviposition Surveillance and a Geographic Information System for Planning Anti‐Vectorial Measures,” American Journal of Tropical Medicine and Hygiene 107, no. 4 (2022): 916–924, 10.4269/ajtmh.21-0364. 36037864 PMC9651510

[72] D. M. Parker , C. Medina , J. Bohl , et al., “Determinants of Exposure to Aedes Mosquitoes: A Comprehensive Geospatial Analysis in Peri‐Urban Cambodia,” Acta Tropica 239 (2023): 106829, 10.1016/j.actatropica.2023.106829. 36649803

[73] F. Wang , Y. Zhu , H. Zhang , et al., “Spatial and Temporal Analyses of the Influences of Meteorological and Environmental Factors on *Aedes albopictus* (Diptera: Culicidae) Population Dynamics During the Peak Abundance Period at a City Scale,” Acta Tropica 245 (2023): 106964, 10.1016/j.actatropica.2023.106964. 37307888

[74] M. Saadatian‐Elahi , M. Rabilloud , T. W. R. Möhlmann , et al., “Effectiveness of Integrated Vector Management on the Incidence of Dengue in Urban Malaysia: A Cluster‐Randomised Controlled Trial,” Lancet Infectious Diseases 25 (2025): 00086–6, 10.1016/S1473-3099(25)00086-6. 40373783

[75] S. Li , H. Tao , and Y. Xu , “Abiotic Determinants to the Spatial Dynamics of Dengue Fever in Guangzhou,” Asia‐Pacific Journal of Public Health 25 (2011): 239–247, 10.1177/1010539511418819. 21852418

[76] H. Padmanabha , D. Durham , F. Correa , M. Diuk‐Wasser , and A. Galvani , “The Interactive Roles of *Aedes aegypti* Super‐Production and Human Density in Dengue Transmission,” PLoS Neglected Tropical Diseases 6, no. 8 (2012): e1799, 10.1371/journal.pntd.0001799. 22953017 PMC3429384

[77] S. Yin , J. Hua , C. Ren , et al., “Spatial Pattern Assessment of Dengue Fever Risk in Subtropical Urban Environments: The Case of Hong Kong,” Landscape and Urban Planning 237 (2023): 104815, 10.1016/j.landurbplan.2023.104815.

[78] A. P. Siqueira , H. L. F. Praça , J. P. C. Santos , et al., “ArboAlvo: Stratification Method for Territorial Receptivity to Urban Arboviruses,” Revista de Saúde Pública 56 (2022): 39, 10.11606/s1518-8787.2022056003546. 35649086 PMC9126578

[79] L. Zheng , H. Y. Ren , R. H. Shi , and L. Lu , “Spatiotemporal Characteristics and Primary Influencing Factors of Typical Dengue Fever Epidemics in China,” Infectious Diseases of Poverty 8, no. 1 (2019): 24, 10.1186/s40249-019-0533-9. 30922405 PMC6440137

[80] J. B. Z. Zahouli , B. G. Koudou , P. Müller , D. Malone , Y. Tano , and J. Utzinger , “Urbanization Is a Main Driver for the Larval Ecology of Aedes Mosquitoes in Arbovirus‐Endemic Settings in Southeastern Côte D'ivoire,” PLoS Neglected Tropical Diseases 11, no. 7 (2017): e0005751, 10.1371/journal.pntd.0005751. 28704434 PMC5526600

[81] M. d. C. Marquetti , M. Castillo , I. Peraza , et al., “ *Aedes albopictus* (Skuse) Dispersion in Havana City, Cuba, 1995–2018,” Acta Tropica 240 (2023): 106839, 10.1016/j.actatropica.2023.106839. 36669694

[82] D. Panda , R. S. Pandit , B. Sahu , R. Kamaraju , and T. K. Barik , “Understanding Mosquito Faunal Diversity: An Approach to Assess the Burden of Vector‐Borne Diseases in Three Representative Topographies (Rural, Urban, and Peri‐Urban) of Ganjam District in Odisha State, India,” Journal of Tropical Medicine 2024 (2024): 9701356, 10.1155/2024/9701356. 39372239 PMC11455597

[83] D. C. P. Câmara , C. T. Codeço , T. Ayllón , et al., “Entomological Surveillance of Aedes Mosquitoes: Comparison of Different Collection Methods in an Endemic Area in Rio de Janeiro, Brazil,” Tropical Medicine and Infectious Disease 7, no. 7 (2022): 114, 10.3390/tropicalmed7070114. 35878126 PMC9324765

[84] A. Hendy , N. F. Fé , I. Pedrosa , et al., “Forest Edge Landscape Context Affects Mosquito Community Composition and Risk of Pathogen Emergence,” iScience 28, no. 1 (2025): 111576, 10.1016/j.isci.2024.111576. 39868037 PMC11758831

[85] A. Leandro and R. Maciel‐de‐Freitas , “Development of an Integrated Surveillance System to Improve Preparedness for Arbovirus Outbreaks in a Dengue Endemic Setting: Descriptive Study,” JMIR Public Health and Surveillance 10 (2024): e62759, 10.2196/62759. 39588736 PMC11611802

[86] J. A. Uelmen , A. Clark , J. Palmer , et al., “Global Mosquito Observations Dashboard (GMOD): Creating a User‐Friendly Web Interface Fueled by Citizen Science to Monitor Invasive and Vector Mosquitoes,” International Journal of Health Geographics 22, no. 1 (2023), 22–28, 10.1186/s12942-023-00350-7. 37898732 PMC10612222

[87] F. G. F. Rios , V. A. do Nascimento , F. G. Naveca , D. S. Vieira , and G. R. Julião , “Arbovirus Detection in Synanthropic Mosquitoes From the Brazilian Amazon and in Mosquito Saliva Using Flinders Technology Associates Cards,” Microbes and Infection 25 (2023), 105046, 10.1016/j.micinf.2022.105046. 36167274

[88] N. H. Rose , M. Sylla , A. Badolo , et al., “Climate and Urbanization Drive Mosquito Preference for Humans,” Current Biology 30, no. 18 (2020): 3570–3579.e6, 10.1016/j.cub.2020.06.092. 32707056 PMC7511451

[89] M. F. Olson , M. L. Ndeffo‐Mbah , J. G. Juarez , et al., “High Rate of Non‐Human Feeding by *Aedes aegypti* Reduces Zika Virus Transmission in South Texas,” Viruses 12, no. 4 (2020): 453, 10.3390/v12040453. 32316394 PMC7232486

[90] N. A. M. H. Abdullah , N. C. Dom , S. A. Salleh , H. Salim , N. Precha , and R. Dapari , “Spatiotemporal Dynamics of Dengue Hotspots in an Urbanizing Landscape: A Five‐Year Analysis in Selangor, Malaysia,” Clinical Epidemiology and Global Health 32 (2025): 101966, 10.1016/j.cegh.2025.101966.

[91] C. K.‐C. Ng , S. Linus‐Lojikip , K. Mohamed , and H. S. S. Amar‐Singh , “Application of Medical Information System to Identify Dengue Outbreak Factors: Insights From a Hyperendemic City in Malaysia,” International Journal of Medical Informatics 177 (2023): 105162, 10.1016/j.ijmedinf.2023.105162. 37549500

[92] M. Khan , M. Pedersen , M. Zhu , H. Zhang , and L. Zhang , “Dengue Transmission Under Future Climate and Human Population Changes in Mainland China,” Applied Mathematical Modelling 114 (2023): 785–798, 10.1016/j.apm.2022.10.027.

[93] S. Knoblauch , M. Su Yin , K. Chatrinan , et al., “High‐Resolution Mapping of Urban *Aedes aegypti* Immature Abundance Through Breeding Site Detection Based on Satellite and Street View Imagery,” Scientific Reports 14, no. 1 (2024): 18227, 10.1038/s41598-024-67914-w. 39107395 PMC11303731

[94] F. Gunay , A. Yildirim , E. Zangaladze , et al., “Predicting the Potential Distribution of *Aedes albopictus* in the Black Sea Region at the Range Edge,” Acta Tropica 267 (2025): 107661, 10.1016/j.actatropica.2025.107661. 40393535

[95] M. Zhao , X. Ran , D. Xing , et al., “Population Genetics of *Aedes albopictus* in the Port Cities of Hainan Island and Leizhou Peninsula, China,” Infection, Genetics and Evolution 117 (2024): 105539, 10.1016/j.meegid.2023.105539. 38104852

[96] T. Tu , J. Yang , H. Xiao , et al., “Spatiotemporal Analysis of Imported and Local Dengue Virus and Cases in a Metropolis in Southwestern China, 2013–2022,” Acta Tropica 257 (2024): 107308, 10.1016/j.actatropica.2024.107308. 38945422

[97] I. Unlu , A. Farajollahi , S. P. Healy , et al., “Area‐Wide Management of *Aedes albopictus*: Choice of Study Sites Based on Geospatial Characteristics, Socioeconomic Factors and Mosquito Populations,” Pest Management Science 67 (2011): 965–974, 10.1002/ps.2140. 21452166

[98] I. Unlu , M. A. Baker , N. Indelicato , D. Drews , Z. Zeng , and R. Vaidyanathan , “Nighttime Applications of Two Formulations of Pyrethroids Are Effective Against Diurnal *Aedes albopictus* ,” Journal of the American Mosquito Control Association 34 (2018): 158–162, 10.2987/17-6720.1. 31442153

[99] S. Ateutchia‐Ngouanet , F. Nanfack‐Minkeu , K. Mavridis , et al., “Monitoring *Aedes* Populations for Arboviruses, *Wolbachia*, Insecticide Resistance and Its Mechanisms in Various Agroecosystems in Benin,” Acta Tropica 253 (2024): 107178, 10.1016/j.actatropica.2024.107178. 38461924

[100] J. V. Fay , S. L. Espinola , M. V. Boaglio , et al., “Pyrethroid Genetic Resistance in the Dengue Vector ( *Aedes aegypti* ) in Posadas, Argentina,” Frontiers in Public Health 11 (2023): 1166007, 10.3389/fpubh.2023.1166007. 37181710 PMC10174043

[101] J. Kerdsawang , A. Ahebwa , R. Ngoen‐Klan , J. Hii , and T. Chareonviriyaphap , “ *Aedes albopictus* Responses to Transfluthrin‐Impregnated Polyester Fabric in a Semi‐Field System at Different Time Periods,” Acta Tropica 264 (2025): 107596, 10.1016/j.actatropica.2025.107596. 40139549

[102] C. A. Ruggerio , G. A. Querejeta , K. B. Conicelli , and R. J. Lombardo , “Integration of Municipal State, Society and University Efforts for Sanitary Risk Prevention Associated With *Aedes aegypti* Mosquito in the Metropolitan Area of Buenos Aires, Argentina,” Tropical Medicine & International Health 26 (2021): 789–799, 10.1111/tmi.13581. 33813766

[103] V. Rusanganwa , B. Bainda , Y.‐D. Gwon , et al., “Evidence of Dengue Virus Exposure and Associated Risk Factors in Rwanda,” IJID One Health 6 (2025): 100056, 10.1016/j.ijidoh.2025.100056.

[104] N. D. Burkett‐Cadena , D. Fish , S. Weaver , and A. Y. Vittor , “Everglades Virus: An Underrecognized Disease‐Causing Subtype of Venezuelan Equine Encephalitis Virus Endemic to Florida, USA,” Journal of Medical Entomology 60, no. 6 (2023): 1149–1164, 10.1093/jme/tjad070. 37862065 PMC10645373

[105] J. W. P. Silva , “Vírus Mayaro: Desafios e perspectivas na saúde pública das Américas,” Brazilian Journal of Implantology and Health Sciences 6, no. 7 (2024): 21–35, 10.36557/2674-8169.2024v6n7p21-35.

[106] I. Lowy , “Leaking Containers: Success and Failure in Controlling the Mosquito *Aedes aegypti* in Brazil,” American Journal of Public Health 107, no. 4 (2017): 517–524, 10.2105/AJPH.2017.303652. 28207332 PMC5343710

[107] J. S. Mackenzie and M. Jeggo , “The One Health Approach—Why Is It So Important?,” Tropical Medicine and Infectious Disease 4, no. 2 (2019): 88, 10.3390/tropicalmed4020088. 31159338 PMC6630404

[108] C. Robbiati , A. Milano , S. Declich , and M. G. Dente , “One Health Prevention and Preparedness to Vector‐Borne Diseases: How Should We Deal With a Multisectoral, Multilevel and Multigroup Governance?,” One Health Outlook 6 (2024): 21, 10.1186/s42522-024-00114-8. 39482757 PMC11529247

[109] H. S. Tiffin , J. R. Gordon , and K. C. Poh , “One Health, Many Approaches: Integrated Vector Management Strategies Support One Health Goals,” Frontiers in Insect Science 5 (2025): 1549348, 10.3389/finsc.2025.1549348. 40530168 PMC12171957

