# Supplementary material for: Climatic, Socioecological and Environmental Determinants of Aedes spp. Dynamics at the Community Interface: A Systematic Review With Reflections From a One Health Perspective
Source: Trop Med Int Health. 2026 Mar 24;31(7):807–21. doi: 10.1111/tmi.70131 (PMC13331526; doi:10.1111/tmi.70131)
Supplement: Supplementary file 1 — Supporting Information S1: Full Search Strategy and Search Log. [file TMI-31-807-s003.docx]

Supplementary Material 1 — Full Search Strategy and Search Log

| S1.1 | Protocol and Reporting Standard | This systematic review was developed and reported according to the PRISMA 2020 statement. The protocol was registered at the Open Science Framework (OSF) under registration code 5mfv6. |
| --- | --- | --- |
| S1.2 | Databases and Search Dates | Searches were carried out in the following electronic databases:   - MEDLINE via PubMed (searched on June 3, 2025) - SciELO (searched on June 9, 2025)   The search period covered studies published from January 2000 to June 2025. No language restrictions were applied. Articles published in English, Portuguese, French, or Spanish were eligible |
| S1.3 | Search Concepts and PRISMA Logic Model | **Concept 1 — Population / Vector** *Aedes spp.* mosquito and related descriptors (e.g., *Aedes aegypti*, *Aedes albopictus*)  **Concept 2 — Determinants / Exposures** Environmental and climatic determinants (temperature, rainfall, humidity, land use), and socio-ecological determinants (urbanization, sanitation, socioeconomic conditions, social determinants)  **Concept 3 — Outcomes / Dynamics** Persistence, survival, reproduction, abundance, population dynamics, larval development, and ecological dynamics  **Concept 4 — One Health / Integrated perspective (filtering conceptual component)**  One Health **/** EcoHealth **/** interdisciplinary or multisectoral integration. |
| S1.4 | Full Electronic Search Strategies (Verbatim) | **Database: PubMed (MEDLINE)** Platform: National Library of Medicine Date searched: June 3, 2025 Search mode: Advanced Search Builder Search field: “All Fields” (default) Publication date filter: 2000/01/01 to 2025/06/03 Other filters applied: None (no language filters) Records retrieved: [n = 969]  PubMed Search String (Complete Boolean Strategy)  ( "Aedes"[All Fields]  OR "mosquito"[All Fields]  OR "Aedes aegypti"[All Fields]  OR "Aedes albopictus"[All Fields])  AND  ( “environment"[All Fields]  OR "climate"[All Fields]  OR "temperature"[All Fields]  OR "humidity"[All Fields]  OR "rainfall"[All Fields]  OR "precipitation"[All Fields]  OR "land use"[All Fields]  OR "urban*"[All Fields]  OR "sanitation"[All Fields]  OR "socioeconomic"[All Fields]  OR "ecological"[All Fields]  OR "social determinants"[All Fields])  AND  ("reproduction"[All Fields]  OR "survival"[All Fields]  OR "persistence"[All Fields]  OR "population dynamics"[All Fields]  OR "larval development"[All Fields]  OR "vector ecology"[All Fields]  OR "abundance"[All Fields]  OR "density"[All Fields])  AND  ( "One Health"[All Fields]  OR "ecohealth"[All Fields]  OR "interdisciplinary"[All Fields]  OR "multisectoral"[All Fields]  OR "integrated"[All Fields]**)** |
| S1.4 | Full Electronic Search Strategies (Verbatim) | **Database: SciELO** Platform: Scientific Electronic Library Online Date searched: June 9, 2025 Search field: “All indexes / All fields” (default SciELO search) Publication period filter: 2000–2025 (when available) Other filters applied: None Records retrieved: [n = 38]  SciELO Search String (Complete Boolean Strategy) |
|  |  | "Aedes" AND ("ambiente" OR "clima" OR "temperatura" OR "umidade" OR "chuva" OR  "urbanização" OR "saneamento" OR "solo" OR "ecologia" OR "fatores" OR "condições") AND ("reprodução" OR "sobrevivência" OR "proliferação" OR "presença" OR "abundância" OR "persistência") |
| S1.5 | Search Documentation and Management (PRISMA transparency) | S1.5.1 Reference management  All retrieved references were compiled in Zotero. Duplicate records across databases were automatically flagged and subsequently verified manually.  S1.5.2 Screening workflow  The selection process followed three sequential steps:   1. Title screening 2. Abstract screening 3. Full-text assessment   Screening was conducted independently by two reviewers, and disagreements were resolved by consensus or adjudication by a third reviewer when needed. |
| S1.6 | PRISMA Flow Inputs (Search Results and Selection Counts) | The PRISMA 2020 flow diagram was built using the counts below:  **S1.6.1 Identification**   - Records identified from PubMed: [n = 969] - Records identified from SciELO: [n = 38] - Total records identified: [n = 1007]   **S1.6.2 Duplicate removal**   - Duplicates removed (Zotero): [n = 35] - Records after duplicates removed: [n = 972]   **S1.6.3 Screening**   - Records screened (title/abstract): [n = 972] - Records excluded at title/abstract stage: [n = 847]   **S1.6.4 Eligibility**   - Full-text articles assessed for eligibility: [n = 125] - Full-text articles excluded, with reasons: [n = 27]   - Not addressing *Aedes* ecology/dynamics: [n = 10]   - Clinical-only focus without vector determinants: [n = 4]   - Not original research (e.g., commentary/letter/abstract): [n = 3]   - Laboratory studies without environmental simulation: [n = 10]   - Full text not retrieved after reasonable efforts: [n = 0]   **S1.6.5 Included**   - Studies included in qualitative synthesis: [n = 98] |
| S1.7 | Notes on Reproducibility | The search strategies were designed to maximize sensitivity to identify studies addressing climatic, environmental, and socio-ecological determinants of *Aedes* spp. persistence and population dynamics within an integrated One Health framing. Searches were conducted using database-native syntax, documented verbatim in this Supplementary Material, and executed on the specified dates. |
|  |  |  |
