# Supplementary material for: Climatic, Socioecological and Environmental Determinants of Aedes spp. Dynamics at the Community Interface: A Systematic Review With Reflections From a One Health Perspective
Source: Trop Med Int Health. 2026 Mar 24;31(7):807–21. doi: 10.1111/tmi.70131 (PMC13331526; doi:10.1111/tmi.70131)
Supplement: Supplementary file 2 — Table S2: Influence of Water Quality and Environmental Factors on Mosquitoes of the Genus Aedes. [file TMI-31-807-s002.docx]

Supplementary Table 2. – Influence of Water Quality and Environmental Factors on Mosquitoes of the Genus *Aedes*

| Assessed variable | Results | Reference |
| --- | --- | --- |
| Water Quality | The life cycle of *Ae. aegypti* was evaluated in various water types—raw sewage, treated effluents, rainwater, and dechlorinated water. The mosquito completed its development in all water types: 17.2% in raw sewage and 1.2% in the polishing pond, compared to 64.5% in dechlorinated water (p < 0.05). | Beserra *et al.* (2009) |
|  | Raw sewage was evaluated as an oviposition and developmental site for *Ae. aegypti*. Egg laying did not differ significantly between sewage and distilled water in either choice or no-choice assays (p = 0.3095; p = 0.4848), and the Oviposition Activity Index was positive in both (+0.1246; +0.1048). Larval development was 2–3 days slower in sewage, but adult emergence remained comparable (p = 0.4256). | Chitolina *et al*. (2016) |
|  | Water quality, plant detritus, and flooding events shape the nutrient composition of *Aedes* breeding sites, altering nitrogen (N) and carbon (C) levels in adult mosquitoes. Stable isotopes were used to trace these nutrient sources, showing that higher isotope concentrations in adults reflected increased nutrient availability in breeding sites and detritus. Notably, mosquitoes with greater nutrient loads also exhibited higher ZIKV viral loads | Yee *et al.* (2019) |
|  | *Ae. albopictus* prefers temporary habitats with clean, clear, odorless, unpolluted waters such as tree holes, tires, cisterns, and fountains—characterized by good sunlight penetration and low turbidity. It was found exclusively (100%) in temporary habitats and represented 5.71% of all mosquitoes emerged (n = 1,123), being absent from polluted and permanent sites. Although specific water-parameter statistics were not reported, species distribution clearly linked *Ae. albopictus* presence to high-quality water, highlighting the importance of controlling clean water containers for vector management | Lubna *et al.* (2024) |
|  | Higher pH reduces larval and pupal abundance, resulting in smaller adults, whereas increased electrical conductivity raises larval density and body size, likely due to greater nutrient availability. Elevated water temperatures accelerate development and produce smaller adults through shorter growth periods and increased stress. Sun exposure also results in smaller mosquitoes, while shaded or larger-diameter containers support larger adults. Overall, these environmental factors directly influence immature mosquito density and adult body size. | Ouédraogo *et al*. (2022) |
| Oviposition site selection and *Ae. Aegypti’s* offspring performance | Oviposition preference did not correspond to improved larval performance, contradicting the 'oviposition preference–offspring performance' hypothesis. Larval success depended more on detritus quantity and type—particularly fresh leaves—than on container exposure time. Thus, container choice alone does not ensure offspring success, as preferred sites may increase competition and slow development. These findings highlight the need to consider resource quality and density-dependent effects rather than oviposition patterns alone when predicting larval outcomes | Montini & Fischer (2024) |
| PAH bioaccumulation and microbiota shift in *Ae. albopictus* development | Exposure to polycyclic aromatic hydrocarbons (PAHs)—toxic pollutants such as benzo[a]pyrene and benzo[b]fluoranthene—affected Ae. albopictus mainly during the larval stage. PAHs persisted in water for up to 45 days and were significantly bioaccumulated by larvae, but not adults. In larvae, PAH exposure reduced gut microbial diversity and increased PAH-degrading bacteria (e.g., *Comamonadaceae*), causing dysbiosis. Adults showed milder effects, with reduced symbionts such as *Wolbachia* and *Cedecea,* potentially impacting immunity, reproduction, and vector competence. 16S rRNA sequencing revealed higher bacterial diversity in adults. Overall, pollution caused bioaccumulation and gut dysbiosis in larvae, while adults exhibited moderate microbiome alterations without bioaccumulation. | Antonelli *et al*. (2024) |
| Heavy metal levels in wild *Ae. aegypti* and *Ae. albopictus* life stages | In artificial breeding sites of *Ae. aegypti* and *Ae. albopictus*, heavy metals, particularly cadmium (Cd) and nickel (Ni), were found in significant amounts. The breeding water's temperature and pH varied, likely influencing metal bioavailability. Notable bioaccumulation of Cd and lead (Pb) occurred in mosquitoes, while higher environmental concentrations of copper (Cu) and chromium (Cr) correlated with lower accumulation in larvae and pupae. This may indicate tolerance or regulatory mechanisms. | Vargas *et al*. (2025) |
| Assessment of exposure of *Ae. albopictus* to microplastics | *Ae. albopictus* is highly sensitive to microplastics (MPs) in water during its larval stage. At 60 MPs/mL, 37% mortality occurred within 48 hours, significantly higher than the control group (18.3%). Higher concentrations (600 and 6000 MPs/mL) resulted in 78.3% and 100% mortality, respectively. Larval death was primarily caused by physical blockage of the digestive tract by MPs, impairing nutrient absorption, with no evidence of chemical toxicity. | (Griffin *et al.* 2023) |
|  | Exposure to microplastics significantly impairs the vectorial efficiency of Ae. albopictus by reducing the ZIKV transmission rate and population transmission rate—most notably on day 14 post-infection, when transmission dropped from 87.5% in controls to 29.41% (5 µg/mL) and 26.67% (50 µg/mL), and PTR decreased from 70% to 25% and 20% (**, p < 0.01). This reduction appears to result from the combined effects of physical adsorption of viral particles onto microplastics within the midgut and extensive transcriptional alterations in genes linked to immunity, signaling pathways (e.g., Toll, Imd, MAPK, Hedgehog), and energy metabolism. Together, these mechanisms hinder viral dissemination from the midgut to the salivary glands. These findings reveal a paradoxical ecological effect: although microplastic pollution is deleterious to ecosystems, its presence may inadvertently attenuate arbovirus transmission by diminishing mosquito vector competence. | (Li *et al*. 2025) |
| Temporary absence of place for oviposition | When females cannot find suitable egg-laying sites, they retain their eggs for longer, which significantly increases fecundity. An experiment found that females that retained eggs for 8 days laid an average of 100 eggs, a 69% increase compared to those that oviposited immediately post-blood feeding (59 eggs), with this difference being statistically significant (p < 0.05). A strong correlation was observed between retention days and egg count (R² = 0.986, p < 0.05). Other parameters, such as fertility, larval mortality, and adult longevity, were not significantly impacted (p > 0.05). | (Gunathilaka & Ganehiarachchi, 2023) |
| Arboviruses alter *Ae. aegypti* flight | Dengue virus infection significantly alters the flight behavior of *Ae. aegypti* mosquitoes. Infected mosquitoes display longer flight durations (average 59.01 s vs. 22.95 s; p = 0.0135 × 10⁻³) but take fewer flights overall compared to uninfected ones. Although they fly less frequently, they cover a larger flight volume (p = 0.029), which indicates greater spatial movement. However, their flight speed and Euclidean distance do not differ significantly (p = 0.064). These behavioral changes could increase the risk of transmission. | (Javed *et al*. 2024) |
| Effects of artificial night lighting | Exposure to artificial light at night (ALAN) significantly reduced diapause incidence in Ae. albopictus — with decreases of 19.15% (F = 8.748, P = 0.042) in the Beijing strain and up to 74.68% (F = 47.000, P = 0.003) in the Guangzhou strain — while simultaneously accelerating larval and pupal development (e.g., larval duration shortened by 1.66 to 3.00 days; t ranging from –7.917 to –5.199, all P < 0.001) and altering the expression of hundreds of genes involved in metabolic and signaling pathways, as indicated by differential gene expression analysis. | (Liu *et al.* 2024) |
| *Ae. aegypti* resting preferences | Most *Ae. aegypti* were found at intermediate heights (0.75–1.5 m), with significantly higher abundance at this level than in the lower and upper zones (*p < 0.001*), indicating a preference for darker, sheltered areas near human activity. As for preferred rooms, bedrooms (35–39%) and bathrooms (~30%) hosted the highest numbers of mosquitoes, with bedrooms showing significantly greater mosquito abundance (*p < 0.001*). | (Seang-arwut et al. 2023) |
