# Supplementary material for: Climatic, Socioecological and Environmental Determinants of Aedes spp. Dynamics at the Community Interface: A Systematic Review With Reflections From a One Health Perspective
Source: Trop Med Int Health. 2026 Mar 24;31(7):807–21. doi: 10.1111/tmi.70131 (PMC13331526; doi:10.1111/tmi.70131)
Supplement: Supplementary file 3 — Table S3: Influence of Temperature on the Biology, Ecology, and Vector Potential of Mosquitoes of the Genus Aedes. [file TMI-31-807-s004.docx]

Supplementary Table 3. Influence of Temperature on the Biology, Ecology, and Vector Potential of Mosquitoes of the Genus *Aedes*

| Assessed variable | Results | Reference |
| --- | --- | --- |
| Temperature | Oviposition activity was strongly seasonal, showing a significant increase during the hot and humid months of July and August—when mean egg counts reached 68.5 ± 12.4 and 74.3 ± 10.7 eggs per trap, respectively (F = 19.62, P < 0.001)—followed by a marked decline in autumn and complete inactivity throughout winter. | Giatropoulos *et al*. (2012) |
|  | Under laboratory conditions, *Ae. albopictus* lived longer than *Ae. aegypti,* though the latter tolerated a broader temperature range. Optimal survival occurred at ~21°C for *Ae. aegypti* and 20–30°C for *Ae. albopictus*. In the field, mortality increased 4.5-fold for *Ae. aegypti* and 3-fold for *Ae. albopictus* due to predation and disease, reducing temperature effects. | Brady *et al.* (2013) |
|  | Temperature emerged as the strongest predictor of mosquito population dynamics. Daily population growth increased steeply between 20°C and 30°C, which represented the optimal thermal window for *Aedes* expansion. Temperatures above 35°C or below 15°C markedly reduced growth, and heat-dependent mortality increased sharply at extremes, significantly lowering population persistence. The model also confirmed that egg diapause is triggered when temperature falls below 17.4°C combined with photoperiods shorter than 11.6 hours, enabling overwintering survival and rapid population recovery once warmer conditions return. | Erguler *et al.* (2016) |
|  | Temperature directly influences the survival, development, and distribution of *Ae. aegypti* and *Ae. albopictus* mosquitoes. Higher temperatures accelerate their life cycle and increase disease transmission, while *Ae. albopictus* adapts better to colder climates. Due to climate change, both species may expand their habitats into new regions. | Kamal *et al.* (2018) |
|  | The weekly mean temperature was statistically significant (p < 0.05) in relation to the increase in dengue cases, and the outbreaks coincided with the hottest periods of 2013 and 2014 in Singapore. | Massaro *et al.* (2019) |
|  | *A. albopictus* thrives at moderately high temperatures (27–30 °C), which accelerate larval development and extend adult female longevity, while temperatures above 32 °C increase mortality. Moderate light (half-shaded environments) enhances pupation and reproduction, whereas full shade or direct sunlight reduces survival and fecundity. | Cui *et al*. (2021) |
|  | This study monitored *Ae. albopictus in* urban Shanghai (2018–2019) using oviposition traps (MOTs) and light traps (LTs). Population peaks occurred in July of both years—2018 (MPI = 14.08%, 33.2 °C) and 2019 (MPI = 8.28%, 32.9 °C). The number of females captured by LTs correlated significantly with MPI (2018: r = 0.792, p = 0.0334; 2019: r = 0.756, p = 0.0489), indicating a strong temperature–abundance relationship. Spatial autocorrelation was significant only for MOT data (p < 0.001), enabling precise identification of infestation hotspots | Zhou *et al*. (2021) |
|  | *Ae. aegypti* showed peak oviposition in summer, accounting for 81.4% of the 4,193 eggs collected, with a maximum in January (1,013 eggs; 24.3%). Activity was nearly absent in winter, no positive ovitraps in August and no data in September. Summer temperatures (21–35 °C, up to 45 °C) and higher humidity favored oviposition, while cooler winter conditions (8.3–24 °C) suppressed mosquito activity, confirming strong seasonality linked to climate. | Abán Moreyra *et al.* (2022) |
|  | Ambient temperature: very high temperatures in microhabitats exposed to direct sunlight (>29°C) reduced egg density | Musunzaji *et al*. (2023) |
|  | Positive correlation between the presence of *Ae. aegypti* eggs and the average weekly maximum temperature (R 0.46 to 0.71). | Uelmen *et al.* (2023) a |
|  | Significantly influences the development and reproductive traits of *Ae. albopictus*. Under warmer urban conditions, mosquitoes exhibited faster immature development, higher fecundity, more gonotrophic cycles, longer female lifespan, and a higher net reproductive rate compared to those from peri-urban areas | Novianto *et al*. (2023) |
|  | Temperature was correlated with Aedes mosquito abundance; however, for *Ae. vexans* and *Ae. geniculatus*, the relationship was negative (r = –0.309; p = 0.001 and r = –0.365; p < 0.001, respectively). In their native region (Iran), these species peak at milder temperatures around 21 °C, suggesting that higher temperatures reduce their abundance. | Nikookar *et al*. (2023) |
|  | ZIKV transmission occurs temperatures between 23 °C and 34 °C increase the risk of transmission; peak at 29 °C. | Lambrechts *et al*. (2024) |
|  | Ecological niche models performed well (AUC = 0.788 for *Aedes aegypti*; AUC = 0.867 for *Aedes albopictus*), indicating robust predictive capacity. The modeled habitat suitability for both species closely aligned with observed occurrence records and was highest along the eastern coast of Brazil, where environmental conditions and vector presence converge to sustain stable populations. Statistical analyses showed that habitat suitability for *Ae. aegypti* was the single most influential environmental predictor of ZIKV transmission risk, yielding the greatest model gain when used alone in jack-knife tests. The mean temperature of the warmest quarter also contributed positively—transmission peaked within the empirically supported 18–34 °C range—although its isolated contribution to model gain was smaller than that of vector suitability. Overall, the study demonstrates that climate, particularly seasonal heat, together with vector ecological niches, are the dominant statistical drivers shaping modeled Zika transmission patterns, whereas socioeconomic variables such as GDP and population density contributed minimally at the coarse spatial resolution available. | Cunze *el al.* (2019) |
|  | Temperature showed a strong unimodal effect on ZIKV transmission, with suitability peaking near 29 °C and remaining possible between ~23–34 °C. Critical thermal intervals (23–27 °C and 29–31 °C) produced steep increases in R₀, and the climate-only model explained 30% of the variation in seroprevalence across Africa. Although many regions already fall within this thermal window, future warming is projected to shift additional large cities toward the optimal temperature, increasing ZIKV transmission suitability. | Caldwell *et al.* (2024) |
|  | The study projects substantial climate-driven expansions in the geographic suitability of *Ae. aegypti* and *Ae. albopictus* across the Americas throughout the 21st century. Under the high-warming scenario (SSP5–RCP8.5), both species show pronounced increases in the total area classified as highly suitable (>0.5), indicating a strong potential for range expansion into regions that are currently too cool for stable transmission. Mean annual temperature emerged as the dominant environmental predictor for both Aedes species, reflecting the strong thermal sensitivity of their development, survival, and vector competence. The models indicate that warming temperatures will allow these mosquitoes to expand northward into increasingly temperate regions, including areas of the United States and Canada, while maintaining suitability in much of their current tropical and subtropical range. Land-use variables played a comparatively smaller role but still influenced regional-scale habitat changes. Overall, the projections demonstrate that, *Ae. aegypti* and *Ae. albopictus* are likely to become even more widespread under future climate scenarios, sustaining and potentially intensifying the risk of dengue, Zika, and chikungunya transmission across the hemisphere. | Gorris *et al*. (2024) |
|  | Ambient temperature strongly influenced Aedes mosquito abundance, showing a positive correlation with total population (r = +0.8; α = 0.05). *Ae. albopictus* was most abundant from June to November, peaking in July and November with no specimens recorded in January and February, indicating sensitivity to cold. | Lubna *et al.* (2024) |
|  | Environmental conditions in Bangladesh strongly favor the persistence and proliferation of *Ae. aegypti*, contributing to the sustained risk of Chikungunya transmission. This country experiences temperatures suitable for Aedes mosquitoes during approximately nine months each year, providing an extended window for arboviral circulation. These favorable thermal conditions, coupled with rapid urbanization, widespread human-made breeding sites, and prolonged periods of rainfall, create an ecological landscape that supports high vector density and efficient viral transmission. The identification of the E1-K211E mutation—known to enhance viral fitness in Aedes aegypti—further underscores the importance of these climate-driven and urban environmental factors in sustaining transmission potential during the 2024 outbreak. | Nasif *et al.* (2024) |
|  | Temperature exerts a nonlinear influence *on Ae. aegypti’s* life cycle, affecting egg hatching, larval development, and adult survival, which peak at moderate values and decline at thermal extremes. The species’ climatic niche—defined by optimal temperature and rainfall—may expand toward temperate zones under global warming, with projections indicating that London could sustain suitable conditions for *Ae*. *aegypti* for 0–5 months annually by 2100, depending on climate variability. | Kaye *et al.* (2024) |
|  | A strong positive correlation was observed between mosquitoes from the *Aedes* genus density and temperature (r = 0.82, p < 0.01) | Abbasi (2025) |
|  | A HR (Hazard Ratio) of 1.55 was observed for each 1°C temperature increase (p < 0.0001), demonstrating a strong association with elevated outbreak risk. Elevated temperatures accelerate vector development cycles and promote viral replication. | Farooq *et al*. (2025) |
|  | Cyclic temperature (25/18°C and 27/20°C) Water renewal (simulating precipitation)  Relationship with outcome: higher temperatures and water renewal increased egg viability and accelerated development. | Neto & Navarro-Silva (2004) |
|  | Oviposition was positively correlated with precipitation (r = 0.57; p < 0.05). No oviposition occurred at temperatures below 16.5 °C. Eggs hatched following rainfall and when temperatures exceeded 18 °C. Winter is a period of vector inactivity. | Stein *et al.* (2005) |
| Temperature and humidity and/or rainfall | Higher minimum temperatures and elevated relative humidity were positively associated with mosquito presence, especially during the pre-monsoon period. Although humidity was not strongly correlated with vegetation, other sources water source also played a role. | Hayden *et al*. (2010) |
|  | The combination of high temperature, high precipitation and regular rainfall patterns was associated with high peaks in the effective reproduction number, which in the multiscale mathematical model, indicated a higher risk of dengue outbreaks. | Wang *et al*. (2019) |
|  | In China, both the Pearl River Delta (PRD) and Baise-Youjiang Medical area (BYM) present climates favorable for dengue transmission but differ in climatic thresholds. In the PRD, higher transmission occurred at 10–27 °C with 79–83% humidity, while in BYM, outbreaks intensified above 17.8 °C and with 170–190 mm of rainfall per month. | Zheng *et al*. (2019) |
|  | Temperature, humidity, and solar radiation significantly shaped the microclimate and vectorial capacity of *Ae. albopictus*. Minimum microclimate temperature increased with macroclimate temperature (β = 0.7801) and humidity (β = 0.0449), promoting nocturnal activity under warm, humid conditions. Maximum microclimate temperature rose with macroclimate temperature (β = 0.9397) and solar radiation (β = 0.0027) but declined with humidity (β = –0.0507), reflecting thermal stress during hot, dry, sunny periods—mitigated by tree cover. The models explained 61% of the variance in minimum and 75% in maximum temperature. | Wimberly *et al.* (2020) |
|  | Temperature was the main factor controlling dengue transmission, optimal between 21 °C and 32 °C and inhibited below 18 °C or above 32.5 °C. Precipitation affected *Aede*s breeding locally moderate rain promoted proliferation, while heavy rain (>30 mm) reduced larvae. Temperature determined transmission thresholds (R₀ > 1), and each 0.71 mm daily rainfall increase expanded risk areas in suitable climates. | Khan *et al.* (2023) |
|  | *Ae. aegypti* abundance peaked during the hottest months, with temperatures exceeding 32 °C and occasionally rising above 37 °C. Temperature variables—especially 7-, 21-, and 28-day lags—were the strongest predictors of mosquito presence (p < 0.0001). Rainfall variables, including same-day and up to 28-day lags, were also associated with mosquito abundance, though their influence was slightly weaker | Uelmen *et al.* (2023) |
|  | Elevated temperature and high humidity, particularly following the monsoon season, create optimal conditions for *Ae. aegypti* proliferation in Pakistan. The region experiences a humid subtropical climate, with an annual average temperature of 19.6 °C and approximately 145 rainy days per year. These conditions increase the availability of breeding sites—such as stagnant water in containers and urban debris. | Qureshi *et al*. (2023) |
|  | At high altitudes, rising temperatures accelerate the Aedes life cycle and enhance vector proliferation, whereas excessive heat in lowlands reduces populations. Moderate rainfall increases breeding sites, while heavy rain washes away eggs and larvae. Modeling projected changes from −55.3% to +118.9% in infectious female populations under different climate scenarios, highlighting the need for locally adapted control strategies. | Lamy *et al.* (2023) |
|  | Temperatures of 25–30 °C speed up the A*edes* life cycle, boost activity, and enhance dengue and Zika virus replication. High humidity (>60%) and frequent rainfall create breeding sites and extend adult survival. In hot, humid regions, these conditions intensify arboviral outbreaks. | Rios *et al.* (2023) |
|  | Temperature and humidity exerted a strong influence on mosquito presence and persistence across the study region. The district’s warm conditions—reaching mean daily maximum of 32.2°C, with minima decreasing to 16.6°C in winter—combined with consistently high relative humidity (~75% year-round) created favorable conditions for mosquito survival and breeding. These climatic parameters, particularly the sustained humidity and temperature stability during most of the year, supported continuous vector activity, while cooler winter temperatures corresponded with reduced species abundance. | Panda *et al.* (2024) |
|  | The abundance of *Aedes aegypti* increased markedly during the warm and rainy months (March and April), coinciding with significantly higher counts than in November and January (χ² = 10.11, p = 0.02). This seasonal rise was influenced by rainfall patterns—abundance decreased with higher cumulative rainfall 22–28 days prior to sampling (χ² = 5.07, p = 0.02)—and was consistently greater in urban than peri-urban neighbourhoods (approximately two-fold; χ² = 8.60, p < 0.01). Moreover, resting behavior varied between cities, with indoor collections in Portoviejo yielding three times more females than in Quinindé, while outdoor counts remained similar across sites | Ortega-López *et al.* (2024) |
|  | The NS5V357E mutation in DENV-2 enhances viral replication at low temperatures, especially at 20 °C, promoting the virus’s persistence during winter and its spread in subtropical regions. This replication advantage, statistically significant, contributes to the replacement of less adapted strains and is associated with more severe outbreaks. The mutation facilitates infection and viral dissemination in *Ae. aegypti*, even under suboptimal temperature conditions, increasing vector competence and extending the transmission period during winter. | Ko *et al.* (2024) |
|  | Temperature and precipitation as central ecological drivers of *Ae. albopictus* expansion in tropical islands like São Tomé and Príncipe. The mean temperature of the coldest quarter and several precipitation metrics (annual and driest periods) strongly predict mosquito presence. Warm temperatures and abundant rainfall create suitable breeding habitats, promoting vector persistence and spread. Climate change may further expand the ecological niche of this invasive species | Rader *et al*. (2024) |
|  | Moderate annual temperatures (16–18 °C) and high humidity (1,000–1,350 mm rainfall) created ideal mosquito conditions. Populations peaked in August—the hottest, most humid period—followed by dengue outbreaks in September–October, consistent with viral incubation time. Humidity prolonged adult survival and egg viability, showing that even mild climates can sustain epidemics when heat, moisture, and human mobility coincide. | Tu *et al.* (2024) |
|  | The *Ae. albopictus* Adult Index (AI) correlated positively with minimum temperature at a 44-day lag (r = 0.27) and mean temperature at 45 days (r = 0.24). In hotspot DH, total Aedes AI correlated with mean temperature at 52 days (r = 0.32), and *Ae. albopictus* DPTI showed a similar lag (51 days, r = 0.32). Relative humidity had negative effects on virus detection, with DPTI of *Ae. albopictus* and *Ae. aegypti* correlating negatively with mean (r = –0.26 to –0.29) and minimum RH (r = –0.24 to –0.27), but positively with maximum RH (r = 0.20–0.25). Rainfall also influenced indices: *Ae. aegypti* AI correlated positively at a 64-day lag (r = 0.33), and *Ae. albopictus* DPTI at 6 days (r = 0.20). | Abdullah *et al.* (2025) |
|  | In Rio de Janeiro’s tropical urban climate, precipitation, humidity, and temperature strongly influence *Ae. aegypti* population dynamics by determining the formation and persistence of artificial breeding sites. The consistently warm, humid conditions reduce seasonality, enabling continuous egg and larval presence. Modeling indicated that climatic variables combined with landscape features explained up to 72% of egg count variability (pseudo-R² = 0.72; 95% CI: 0.70–0.74) and 74% of larval abundance (pseudo-R² = 0.74; 95% CI: 0.72–0.76). | Knoblauch *et al*. (2025) |
|  | Rainfall had a direct but secondary influence: increases in precipitation expanded available breeding sites and boosted population growth, but the effect was highly volatile and short-lived, especially in urbanized environments where rapid evaporation and drainage reduce water accumulation. The model showed that rainfall effects were density-independent, influencing daily growth rates without long-term stability. | Erguler *et al.* (2016) |
|  | Rainfall exhibited a "wet-dry steps" pattern (regular alternation of wet and dry periods), which preceded outbreaks with high linearity (R^2^ >0.99). However, statistical analyses found no significant correlation between monthly rainfall and dengue cases (R^2^ = 0.0021), suggesting that temporal regularity of rainfall may be more relevant than total volume. | Ng *et al.* (2023) |
|  | Rainfall directly influences *Ae. aegypti* populations by creating breeding sites in containers with stagnant water, increasing larval survival by up to 60%. Dengue cases typically rise 45–75 days after peak rainfall, matching mosquito and viral incubation cycles. The rainfall–outbreak correlation is stronger in regions with distinct seasons and weaker where rain is constant year-round. | Oliveira *et al.* (2023) |
| Humidity and/or rainfall (precipitation) | In Recife (2001–2019), increased rainfall promoted *Ae. aegypti* breeding and higher dengue cases, mainly from March to July. Yet, four of six major outbreaks (2002, 2012, 2015, 2016) occurred in drier months due to new serotype introductions (DENV-3, DENV-4). Models using only weather data showed weak correlation (r = 0.37), but adding serotype data greatly improved it (r = 0.88), with precipitation and all serotypes significant predictors. | Borges *et al.* (2024) |
|  | In Malaysia, dengue hotspots were concentrated in rainy seasons (November–March and June–September), coinciding with higher precipitation. Spatial analyses (IDW, Getis-Ord Gi*) revealed significant transmission clusters (p < 0.01) in densely populated areas during wet months, indicating that increased rainfall promotes *Aedes* breeding and raises dengue risk | Abdullah *et al.* (2025) |
|  | The reduced total rainfall in the semi-arid region of Tehran, *Ae. aegypti* populations increased due to greater reliance on artificial breeding sites. The analysis revealed that years with more rainy days were associated with dengue outbreaks, but the most critical factor was the accumulation of water in urban containers. The negative correlation between total rainfall and mosquito density (r = −0.68; p < 0.05) indicates that, in the absence of consistent rainfall, the vector depends more on artificial water sources for oviposition, particularly in urban areas. | Abbasi (2025) |
|  | Low humidity conditions induce significant physiological and behavioral changes in *Ae. aegypti*, resulting in an increased propensity for multiple blood feedings within a single gonotrophic cycle. In dry environments (30–40% RH), mosquitoes took on average two to three additional blood meals before oviposition—a statistically significant increase compared to those kept in humid conditions (p < 0.05) | Holmes *et al.* (2025) |
