# Supplementary material for: Climatic, Socioecological and Environmental Determinants of Aedes spp. Dynamics at the Community Interface: A Systematic Review With Reflections From a One Health Perspective
Source: Trop Med Int Health. 2026 Mar 24;31(7):807–21. doi: 10.1111/tmi.70131 (PMC13331526; doi:10.1111/tmi.70131)
Supplement: Supplementary file 4 — Table S4: Anthropogenic Factors Influencing the Reproduction, Viability, and Persistence of Mosquitoes of the Genus Aedes. [file TMI-31-807-s001.docx]

Supplementary Table 4 - Anthropogenic Factors Influencing the Reproduction, Viability, and Persistence of Mosquitoes of the Genus *Aedes*

| Assessed variable | Results | Reference |
| --- | --- | --- |
| Social vulnerability | Dengue cases occurred mainly in middle-class areas with higher income (p < 0.01), greater education (p < 0.01), fewer residents per household (p < 0.01), and better sewage infrastructure (p < 0.01), reflecting imported infections among more mobile individuals. In contrast, mosquito presence was greater in areas with more houses (p < 0.01), larger households (p < 0.01), and broader water (p < 0.05) and sewage coverage (p < 0.01), independent of income. | Barcellos *et al*. (2005) |
|  | Socioeconomic stratum: Inverse relationship with infestation — the lower the stratum, the greater the presence of vectors. Higher stratum had a lower chance of positivity (OR = 0.4). Education level: Having at least one year of schooling was a protective factor against the presence of immature forms (adjusted OR = 0.3). | Quintero *et al.* (2009) |
|  | Socioeconomic stratum influenced *Aedes* mosquito populations and dengue incidence mainly through population density and residential areas per capita. Population density showed a positive correlation with dengue cases (ρ = 0.31; p < 0.05), while residential area per capita correlated negatively (ρ = –0.27; p < 0.05), indicating that higher crowding increases transmission risk, whereas greater living space reduces it. | Li *et al.* (2011) |
|  | A significant positive correlation between poverty levels and *Ae. albopictus* abundance (R² = 0.53, p = 0.026), indicating higher infestation in poorer areas (New Jersey, USA). This was determined through linear regression using data from BGS traps | Unlu *et al.* (2011) |
|  | Traditionally associated with vegetated areas, *Ae. albopictus* was found up to 400 meters away from green zones, indicating its ability to thrive in neglected urban environments. Poor housing conditions—such as inadequate sanitation, irregular water supply, and high population density (138 inhabitants/km²)—facilitated its establishment. The mosquito was frequently detected in key sites (e.g., junkyards, repair shops) and coexisted with *Ae. aegypti* in 84.6% of locations. Its presence indoors (in households and schools) suggests a shift toward domestication (Rio de Janeiro, Brazil) | Ayllón *et al. (*2018) |
|  | Socioeconomic factors explained 54.1% of dengue incidence in the Pearl River Delta (PRD) and 56.6% in the Yunnan–Myanmar Border (BYM). In PRD, population density (20.8%) and urban land ratio (18.6%) were the main contributors, while in BYM, urban land ratio (30.1%) and road density (19.6%) were the strongest predictors. The models had strong fit (R² = 0.834 in PRD; R² = 0.973 in BYM), indicating that urbanization, infrastructure, and population concentration strongly influence dengue transmission. | Zheng *et al*. (2019) |
|  | Socioeconomic variables were essential in constructing the vulnerability index, grouped into three main factors: low socioeconomic status (including low income, low education, and precarious housing), presence of children, and suburban characteristics. These variables were extracted from the 2016 census and analyzed using principal component analysis (PCA). The presence of the mosquito vector was modeled based on environmental and urban data, revealing that areas with moderate vegetation, high building density, and public residential land use had a higher probability of harboring the mosquito. The overlap between areas of high vulnerability and high vector presence led to the identification of 17 high-risk hotspots. | Pedro *et al.* (2022) |
|  | Urban density significantly increased *Ae. aegypti* proliferation by creating favorable microhabitats in densely built areas such as informal settlements and compact neighborhoods. These sites showed the highest environmental suitability for eggs and larvae, confirmed by strong correlations between suitability maps and ovitrap data (r = 0.76 for larvae; p ≤ 0.05). Fine-scale spatial heterogeneity of the urban landscape explained abrupt variations in mosquito presence, emphasizing the need for locally adapted vector control strategies. | Knoblauch *et al*. (2025) |
|  | The average income of household heads was inversely associated with chikungunya incidence: lower income areas had a higher number of cases. The Relative Risk (RR) of 0.86 (95% CI: 0.83–0.90) (Salvador, Brazil). | Argibay *et al.* (2025) |
|  | Areas with higher vulnerability had significantly higher dengue infection rates, especially in Sri Lanka (IRR = 1.97; 95% CI: 1.08–3.65) and Thailand (IRR = 14.56; 95% CI: 5.55–39.90). During the hotter months, the risk increased even further: in August, Sri Lanka had an IRR = 6.11 (95% CI: 2.83–13.47), and in June, Thailand showed an IRR = 23.61 (95% CI: 9.39–61.67). | Wang *et al.* (2025) |
|  | Rapid urbanization and inadequate infrastructure are key factors in the spread of *Ae. aegypti*, leading to increased dengue transmission. Statistical model indicated that housing density within 25 meters is a strong predictor of high mosquito abundance (OR = 68.99; p < 0.001). Water-holding containers linked to poor drainage and cumulative rainfall also contribute to this issue (OR = 1.004; p < 0.001). Areas with unplanned growth and low income, marked by poor sanitation, face a higher risk of infestation. The overlap of *Ae. aegypti* and *Ae. mediovittatus* is more common in densely populated peri-urban areas with abundant vegetation (OR = 46.15 and OR = 1.008, respectively; both p < 0.001). | (Little *et al.* 2011) |
|  | Water supply regularity proved to be a critical factor in *Aedes* proliferation. The study found that 21.6% of rural households experienced irregular water supply, compared to only 8.3% in the urban area, a statistically significant difference (p < 0.05). This irregularity leads residents to store water in containers, increasing the number of potential breeding sites. This is reflected in the higher entomological indices observed in the rural area: the Household Index (HI) was 22.5%, the Container Index (CI) was 6.5%, and the Breteau Index (BI) reached 40.7, all higher than those in the urban area (HI = 4.4%, CI = 0.9%, BI = 6.1). | (Singh *et al.* 2019) |
|  | The study demonstrated that the spatial risk of dengue in Hong Kong is primarily driven by environmental and urban factors that favor Aedes albopictus presence. The optimal MaxEnt model (AUCTRAIN = 0.847; AUCTEST = 0.841) identified NDVI (31.8% permutation importance), the frontal area index—FAI (22.8%), and the aggregation of public and private residential land (17%) as the strongest predictors of mosquito habitat suitability, underscoring that areas with higher concentrations of residents, dense housing structures, and medium vegetation coverage were the most conducive to vector presence. These same areas corresponded to regions of elevated vulnerability: spatial analysis revealed significant clustering of high hazard–high exposure grids (global Moran’s I = 0.563, z = 162.6), with 14.2% of the city’s grids falling into this category. Principal component analysis further showed that socioeconomic disadvantage, high proportions of children, and suburban occupational profiles explained 75% of the variance in population vulnerability. When hazard, exposure, and vulnerability were integrated, 17 LTPUs emerged as the highest-risk areas (risk index > 0.72), largely concentrated in eastern Hong Kong Island, Kowloon, and three northern hotspots—precisely where dense populations and residential aggregations align with environmental suitability for Aedes albopictus. | Yin *et al.* (2023) |
|  | In the urban environment, *Ae.aegypti* was significantly more abundant than *Ae. albopictus,* as confirmed by a Fisher’s exact test (p = 0.0039). This distribution reflects *Ae. aegypti’s* stronger adaptation to domestic environments. | Câmara *et al*. (2022) |
| Urban infrastructure | While improved sanitation is generally expected to reduce vector-borne diseases, the findings showed that as the percentage of piped water and sewage coverage increased, so did the correlation between rainfall and dengue incidence. Specifically, in cities with better sanitation indicators (cluster C2), the Pearson correlation between piped water coverage and the cross-correlation coefficient (DCCAC) was ρ = 0.679 (p < 0.05), and the correlation with sewage coverage was even stronger, at ρ = 0.829 (p < 0.05). (Brazil) | Oliveira *et al*. (2023) |
|  | Abandoned irrigation ditches in San Juan, Argentina, have become productive breeding sites for Aedes aegypti. These cement-lined ditches, filled with stagnant water and waste, exhibited favorable physicochemical conditions for larval development—conductivity of 772 μS/cm, total dissolved solids of 552 ppm, salinity of 380 ppm, and pH 7.7. Ditches positive for Aedes showed significantly higher salinity (p = 0.02), pH (p = 0.01), and conductivity (p = 0.02) than negative ones. Larvae successfully developed into adults despite the arid environment, demonstrating the mosquito’s ecological adaptability and underscoring the need to manage neglected urban infrastructures to prevent disease outbreaks. | Illa *et al.* (2024) |
|  | Urban areas with low- and middle-cost housing showed greater vulnerability to dengue due to high population density and limited infrastructure, resulting in more cases and weaker vector control. However, clusters that received integrated vector management (IVM) interventions had significant reductions in dengue incidence during outbreaks (RR = 0.71; 95% CI: 0.51–0.98; p = 0.036) and in prolonged hotspots lasting over 30 days (RR = 0.57; 95% CI: 0.35–0.93; p = 0.025). | Saadatian-Elahi *et al*. (2025) |
|  | The analysis revealed a strong correlation between urbanization and dengue hotspot persistence, with the overwhelming majority of the 3,046 geocoded hotspots concentrated in highly urbanized, high-population-density districts. Specifically, Petaling (1,172 hotspots; population density 4,755/km²), Hulu Langat (828 hotspots; 1,673/km²), Klang (414 hotspots; 1,708/km²), and Gombak (417 hotspots; 1,452/km²) were identified as the primary loci of sustained transmission over the five-year study period. In stark contrast, rural districts with significantly lower population densities, such as Sabak Bernam (4 hotspots; 107/km²) and Kuala Selangor (14 hotspots; 240/km²), reported minimal hotspot activity. This spatial clustering underscores high population density and the associated urban environment—characterized by inadequate waste management and abundant artificial water-holding containers—as a paramount driver of dengue transmission risk in Selangor. | Abdullah *et al.* (2025) |
|  | Deficiencies in urban infrastructure, such as irregular waste collection and limited access to piped water, contribute to increased presence of *Ae. aegypti.* Accumulated waste creates breeding sites, while stored water in open containers serves as oviposition sites. These factors are significant predictors of egg and larval abundance (p ≤ 0.05). Digital imagery object detection achieved F1-scores ≥ 0.84 for identifying risk areas. Combining these environmental indicators with entomological data enhances the assessment of urban vulnerability, guiding public health interventions. | Knoblauch *et al.* (2025) |
|  | *Ae. albopictus* was recorded 44 times in tires, 12 times in bamboo traps at ground level, and 4 times at two meters in height, demonstrating broad ecological plasticity and adaptation to both natural and artificial environments. *Ae. aegypti* was found exclusively in tires, in five collections, always cohabiting with other species. The sylvatic species *Ae. terrens* showed a significant preference for tires over other breeding sites (p < 0.05) | Zequi *et al.* (2005) |
|  | Lack of piped water was significantly associated with higher *Ae. aegypti* presence. In Mexico, 39% of households lacked piped water and stored water in uncovered or poorly sealed containers, creating breeding sites. Logistic regression indicated that having piped water was protective (OR = 0.12, 95% CI: 0.02–0.73 p = 0.021;), meaning households with piped water were 88% less likely to harbor *Ae. aegypti* than those without. | Hayden *et al.* (2010) |
|  | Drums, tires, cement tanks, metal containers, and earthen pots were the main breeding sites for *Ae. aegypti* in both urban and rural areas, with higher positivity in peridomestic containers. Mosquito presence was significantly greater in containers with rainwater (8.8%) than with tap water (1.9%) (p < 0.05), and in unused containers (10.7%) compared to those used daily (0.5%) (p < 0.05).. | Singh *et al.* (2019) |
|  | *Ae. aegypti* as the predominant species, with 55.2% of immature mosquitoes found in containers with no immediate use—such as discarded tires and buckets often filled with rainwater—located in the yards of households. (Kenya) | (Forsyth *et al*. (2020) |
| Human-made containers serve as artificial breeding grounds for mosquitoes | The density of artificial breeding containers—such as water tanks, tires, plant pots, and trash bins—was one of the strongest predictors of *Ae. aegypti* presence. Detected via computer vision models with high accuracy (F1-score ≥ 0.84), container density was statistically significant (p ≤ 0.05) and, together with other environmental variables, explained up to 75% of larval count variability (pseudo-R² = 0.74; 95% CI: 0.72–0.76) and 73% of egg count variability (pseudo-R² = 0.72; 95% CI: 0.70–0.74) in NB-GLM models. | Knoblauch *et al.* (2024) |
|  | *Ae. aegypti* was present in 85 of the 358 surveyed tires, with a median abundance of 17 individuals per infested tire. The abundance pattern was highly skewed, where a single tractor tire accounted for 18.5% of all *Ae. aegypti* immatures collected, highlighting the role of large tires as key containers. The median abundance of *Ae. aegypti* did not show a significant increasing trend across tire categories (J-T = 1526, p = 0.472). Furthermore, no significant difference in abundance was found between tires in vertical and horizontal positions for this species (U = 879, p = 0.534). These findings underscore that while large tires can be hyper-productive for *Ae. aegypti*, leading to concentrated emergence, the species' infestation is not consistently dependent on tire size in this temperate urban setting | Achaga & Vezzani (2024) |
|  | *Ae. albopictus* was mainly found in shaded artificial containers, such as discarded plastics near vegetation, whereas *Ae. aegypti* predominated in open areas, especially in used tires and discarded containers. The lack of sympatric occurrence indicates clear ecological differentiation between the two species (Kenya). | Babalola *et al.* (2025) |
|  | In urban areas, mosquito abundance was negatively associated with the number of geckos (p < 0.001), indicating a potential predatory role. In rural areas, hanging clothes at intermediate height significantly increased mosquito abundance (p = 0.002), with differences between intermediate and low (p < 0.05) and intermediate and high positions ( p < 0.01). | Seang-arwut *et al.* (2023) |
|  | Between 2013 and 2018, the domiciliation of *Ae. albopictus* in Cuba was evidenced by a stable rise in indoor breeding sites, ranging from 8% to 21.5% (mean = 15%), mainly in household containers such as water tanks, buckets, and kitchen basins. The infestation risk was significantly higher in peri-urban areas (aOR = 10.32; 95% CI: 4.17–25.56) and during 2010–2018 compared to 1995–1999 (aOR = 3.08; 95% CI: 2.02–4.70). | Marquetti *et al.* (2023) |
|  | *Ae. aegypti* was predominant in urban areas, while *Ae. albopictus* showed a broad distribution, being predominant in both urban and peri-urban areas, indicating its adaptation to new urban environments. Among the *Aedes* specimens collected, *Ae. albopictus* accounted for 39.56%, *Ae. aegypti* for 32.94%, and an unidentified *Aedes species* (Aedes n. sp.) for 27.50% | Panda *et al*. (2024) |
|  | Neighborhood type (urban vs. peri-urban) was statistically significant in explaining the abundance of female *Ae. aegypti* mosquitoes. Urban neighborhoods—characterized by higher population density, limited access to piped water, and greater reliance on water storage containers—had approximately twice the mosquito abundance compared to peri-urban areas (p< 0.01) | Ortega-López *et al.* (2024) |
|  | Urban edges had a significantly higher presence of *Ae. albopictus* (p < 0.0001) compared to rural edges and continuous forests, indicating stronger adaptation to human environments. Rural edges hosted both urban and sylvatic species, creating zones of spillover and spillback risk. Mosquito community composition varied significantly across edge types (p < 0.0001), with greater species evenness at rural edges (p = 0.0005) | Hendy *et al.* (2025) |
|  | A positive correlation was observed between the percentage of wooden houses and egg abundance in February (p < 0.05), March (p < 0.05), and May (p < 0.05); a positive correlation with very precarious houses in January (p < 0.05); and a negative correlation with brick houses in April (p < 0.05). Additionally, the presence of water pipes located outside the house but within the property was positively correlated with egg abundance in March (p < 0.05), suggesting that water storage in open containers may promote breeding sites. The variable “unmet basic needs” also showed a positive correlation with egg abundance in February (p < 0.05), indicating that areas with greater social vulnerability tend to offer more favorable environmental conditions for vector proliferation. | Abán Moreyra *et al*. (2022) |
|  | The number of toilets in a household was found to be a statistically significant predictor of increased exposure to *Aedes* mosquito bites, as measured by antibody responses to *Aedes aegypti* salivary gland extract (SGE). Specifically, the Bayesian geostatistical model showed that each additional toilet in the home was associated with a 4.36% increase in the log-transformed antibody response (95IC%2.56-6.19) | Parker *et al*. (2023) |
| Household environment | The study evaluated several socioeconomic variables as potential predictors of female *Aedes aegypti* abundance in the Tampa Bay area, Florida. the age of housing was an important factor: homes built between 1960–69 (p < 0.0001) and 1970–79 (p < 0.0001) was significantly associated with higher mosquito densities. | Uelmen *et al. (*2023) a |
|  | The influence of housing type on the presence of *Ae. aegypti* breeding sites, classifying homes as "kuccha" when they lacked a concrete roof. The proportion of kuccha houses was similar in both urban and rural areas—9.4% and 9.3%, respectively—with no statistically significant difference between the locations (p = 0.97) (India) | Singh *et al.* (2019) |
|  | In rural areas, houses with cement walls had a higher number of mosquitoes (p = 0.014), as did those with bathrooms located outside the residence (p = 0.017). The number of rooms was also positively associated with vector abundance (p = 0.003), suggesting that larger homes offer more resting sites (Thailand). | Seang-arwut *et al.* (2023) |
|  | Housing prices in Shanghai were analyzed as socioeconomic indicators reflecting the density of *Ae. albopictus* populations. The hypothesis posited that lower housing prices, indicating socioeconomic deprivation, correlate with poorer environmental conditions and increased mosquito proliferation. Using Poisson regression and geographically weighted Poisson regression (GWPR) models, the study found a significant negative association between housing prices and mosquito density measured by the Mosquito Oviposition Trap Positivity Index (MPI) across July, August, and September. The GWPR analysis confirmed that housing prices significantly influenced mosquito abundance, particularly in urban fringe areas. | Wang *et al.* (2023) |
|  | The study compared a dengue hotspot (DH) and a non-hotspot area (NDH), revealing clear differences in vegetation cover. In the NDH, most traps were surrounded by vegetation, whereas all traps in the DH were in areas with less than 10% vegetation. Although *Ae. albopictus* density was higher in the NDH, no mosquitoes tested positive for dengue (DPTI = 0%) and no human cases were reported. In contrast, both *Ae. aegypti* and *Ae. albopictus* were found in the DH, with mean DPTIs of 2.54% and 1.93%, respectively, alongside confirmed dengue cases. Significant correlations between the dengue-positive trap index and climatic variables—rainfall (r = 0.31; p < 0.05) and mean temperature (r = 0.30; p < 0.05)—indicate that low vegetation cover and urbanized environments may increase the risk of dengue transmission. | Abdullah, *et al.* (2025) |
|  | Population density was a key socioeconomic factor linked to dengue incidence in Guangzhou, China, showing a significant positive correlation with case numbers (Spearman r = 0.31; p < 0.05). This suggests that in urban areas with a relatively uniform distribution of *Aede*s mosquitoes, higher human density increases contact rates and, consequently, the likelihood of virus transmission. | Li *et al.* (2011) |
|  | Housing density and urban structure were strongly linked to dengue vector presence. *Ae. aegypti* was significantly more common in areas with high housing density (OR = 68.99; p < 0.001), while *Ae. mediovittatus* was less frequent in large, continuous urban zones (OR = 0.13; p < 0.001). Their co-occurrence also increased with housing density (OR = 46.15; p < 0.001). | Little *et al.* (2011) |
|  | In Athens, Greece, ovitrap monitoring over two years showed that more urbanized areas had significantly higher *Ae. albopictus* oviposition activity (p < 0.001), indicating that human population density and urban structure strongly influence the species’ distribution and abundance. | Giatropoulos *et al*. (2012) |
|  | The number of humans per house increased secondary dengue infections (p < 0.001), while more residents in the index household reduced risk (p < 0.001), indicating mosquito aggregation drives super-spreading. The number of biting mosquitoes was the strongest predictor (p < 0.001), and the pupae × humans per house index best predicted epidemic risk (χ² = 1032.3), outperforming pupae per person (χ² = 308.3). | Padmanabha *et al.* (2012) |
|  | Human population density strongly modulated climatic effects: areas with higher human density showed significantly elevated mosquito abundance (β = 0.013, P < 0.001), reflecting the increased availability of artificial containers that act as stable breeding sites. These anthropogenic structures buffered environmental instability, particularly fluctuations in temperature and rainfall—reducing the sensitivity of population growth to climatic variation and allowing mosquito populations to persist even under suboptimal environmental conditions. | Erguler *et al.* (2016) |
|  | Human density within a 20 km radius was a significant factor influencing Ae. aegypti host preference, explaining 18% of variation in odor-driven behavior (likelihood ratio test, p = 1.0 × 10⁻⁵). | Rose *et al.* (2020) |
|  | Housing conditions, socioeconomic status, and urban occupation patterns strongly affect human exposure to mosquito vectors and ZIKV transmission risk. In Texas, widespread use of air conditioning and window screens effectively reduces human-mosquito contact. Conversely, in lower-income neighborhoods with lower population density, the greater presence of animal hosts may divert mosquito feeding away from humans. | Olson *et al.* (2020) |
|  | In Tampa Bay, Florida*, Ae. aegypti* abundance was positively associated with several socioeconomic factors, including human population density, number of housing units, median household income, and proportion of White population (all p < 0.0001). | Uelmen *et al. (2023) a* |
|  | In urban Malaysia, high housing density and informal gardens around homes were strongly linked to greater *Ae. aegypti* presence and increased dengue cases. Dense housing enhanced human–vector contact, while gardens with pots and plants created shaded, humid microhabitats ideal for oviposition. Spatial analyses identified these features in key transmission zones, though no formal p-values were reported. | Ng *et al.* (2023) |
|  | Human population density increases dengue transmission by providing more blood sources and breeding sites for *Ae. albopictus*. Modeling showed that higher density elevates egg recruitment and the basic reproduction number (R₀), with southern and eastern China (R₀ > 1) at greater outbreak risk, while sparsely populated regions remained low risk. | Khan *et al.* (2023) |
|  | Densely populated areas, characterized by greater water storage and waste accumulation, were key predictors of Aedes proliferation. Incorporated into NB-GLM models within a 200 m buffer around ovitraps, population density, along with other variables, explained up to 74% of larval and 72% of egg count variability, showing statistical significance across seasons (p ≤ 0.05). | Knoblauch *et al*. (2024) |
|  | Human population density was the main determinant of environmental suitability for *Ae. albopictus* in the Black Sea region, contributing 62.4% to the Maxent model—far surpassing climatic variables. The model showed strong accuracy (AUC = 0.83). | Gunay *et al.* (2025) |
|  | Higher population density was associated with lower chikungunya incidence (RR = 0.78; 95% CI: 0.77–0.80), suggesting a protective effect in denser, urbanized areas of Salvador, Brazil. These neighborhoods typically have high-rise housing, better infrastructure, and sanitation, which reduce mosquito breeding and human exposure. In contrast, low-density peripheral areas with informal housing and poor services showed greater *Ae. aegypti* proliferation and higher transmission risk. | Argibay *et al.* (2025) |
|  | Mobile phone data from 2 million users in Singapore were used to integrate real commuting patterns into an agent-based model. The model with actual mobility data had the highest temporal prediction accuracy (R² = 0.65), outperforming Lévy (R² = 0.62), radiation (R² = 0.56), and random mobility (R² = 0.51). Spatial predictions were also closest to observed dengue case distributions using real and radiation-based mobility. | Massaro *et al.* (2019) |
| Urban mobility | *Ae. albopictus* populations in southern China showed high haplotype diversity (Hd = 0.807–0.968) but low nucleotide diversity, indicating recent expansion. Genetic analysis revealed three clades unrelated to sampling locations. High gene flow occurred in port and urban areas (Nm > 13) due to human movement, while some rural populations experienced bottlenecks but still maintained gene flow. Overall, extensive genetic exchange limits population differentiation, complicating vector control. | Zhao *et al. (*2024) |
|  | Most imported dengue cases in 2019 came from Southeast Asia (Cambodia, Myanmar, Thailand) and Guangdong, China, and were concentrated in high-mobility districts like Yubei and Wanzhou, which host airports and railway stations. Spatial analysis showed these districts also experienced the highest local outbreak incidence, indicating human mobility strongly influenced virus introduction and spread. | Tu *et al.* (2024) |
|  | Nighttime applications of Duet™ and Anvil® effectively reduced *Ae. albopictus* populations in New Jersey, USA, with Duet™ twice as effective as Anvil® (p < 0.0001) due to prallethrin-induced flight activity. Cage trials showed higher mosquito mortality in front yards (78%) than backyards (58%) (p < 0.001), while spray patterns were similar, indicating efficacy differences were chemical based. | Unlu *et al.* (2018) |
|  | In Benin’s agroecosystems, *Ae. aegypti* was the dominant species (93.9%) and showed significant permethrin resistance in two of three sites (p = 0.02). Knockdown resistance (kdr) mutations (F1534C, V1016G/I, S989P), including double and triple combinations, were present, along with overexpression of P450 detoxification genes (CYP6BB2, CYP9J26, CYP9J32), indicating metabolic resistance. Natural *Wolbachia spp.* was also detected, potentially affecting biological control strategies. | Ateutchia-Ngouanet *et al.* (2024) |
|  | Infrequent temephos application (every three months) was linked to higher *Ae. aegypti* presence (p = 0.024), indicating low larvicide effectiveness. In urban areas, repellent use correlated with more mosquitoes (p = 0.022), likely reflecting perception bias | Seang-arwut *et al.* (2023) |
| Use of repellents or interventions with repellents | In Posadas, Argentina, pyrethroid resistance in *Ae. aegypti* (kdr mutations V1016I and F1534C) was higher in high socioeconomic status (SES) neighborhoods, with 67% of mosquitoes carrying at least one resistance allele, compared to 34% in low SES areas. The fully susceptible genotype (VV/FF) was absent in high SES neighborhoods but accounted for 14% of mosquitoes in low SES areas. Mosquito abundance was also greater in high SES neighborhoods (3.32 vs. 2.16 mosquitoes per household; p < 0.01). | Fay *et al*. (2023) |
|  | The study tested polyester fabrics treated with transfluthrin (TFT-P) against *Ae. albopictus* under semi-field conditions. Treated fabrics significantly reduced mosquito landings (60.7% morning vs. 31.97% afternoon; p < 0.001) and lowered blood-feeding rates (37.5% vs. 67.7% in control; p = 0.004). Egg hatching decreased (31.2% vs. 54.5%; p = 0.039), though egg numbers per female were unchanged. Temperature and humidity also influenced landings (p = 0.009 and p = 0.019). TFT-P fabrics effectively reduce bites and mosquito reproductive potential. | Kerdsawang *et al.* (2025) |
|  | Nighttime applications of Duet™ and Anvil® effectively reduced *Ae. albopictus* populations in New Jersey, USA, with Duet™ twice as effective as Anvil® (p < 0.0001) due to prallethrin-induced flight activity. Cage trials showed higher mosquito mortality in front yards (78%) than backyards (58%) (p < 0.001), while spray patterns were similar, indicating efficacy differences were chemical based. | Unlu *et al.* (2018) |
| Human professional profiles | Farmers had a higher risk of past dengue infection compared to other occupations. Multivariate analysis revealed that office workers (adjusted OR = 0.53; 95% CI: 0.32–0.86), vocational professionals (OR = 0.48; 95% CI: 0.26–0.90), and other occupations (OR = 0.52; 95% CI: 0.34–0.80) had significantly lower odds of DENV IgG seropositivity (p < 0.05). This association is biologically plausible, as farmers are more frequently exposed to outdoor environments where *Ae. aegypti* mosquitoes—the primary dengue vector—are commonly found (Rwanda) | Rusanganwa *et al.* (2025) |
|  | The occupational distribution (work x unnployment, indoor x outdoors; local x imported cases) showed a statistically significant difference between the groups compared (χ² = 99.544; p < 0.05), as did gender (χ² = 58.881; p < 0.05) and age (t = 3.084; p < 0.05) distributions between imported and local cases in 2019. | Tu *et al*. (2024) |
